# Risk of Developing Alzheimer’s Disease and Related Dementias in ALLHAT Trial Participants Receiving Diuretic, ACE-Inhibitor, or Calcium-Channel Blocker with 18 Years of Follow-Up

**Published:** 2022-04-22

**Authors:** Xianglin L. Du, Lara M. Simpson, Mikala C. Osani, Jose-Miguel Yama, Barry R. Davis

**Affiliations:** 1 Department of Epidemiology, Human Genetics and Environmental Sciences, University of Texas Health Science Center at Houston, Houston, USA; 2 Department of Biostatistics and Data Science, University of Texas Health Science Center at Houston, Houston, USA

**Keywords:** Alzheimer’s disease, Dementia, Anti-hypertensive drugs, ALLHAT trial, Medicare claims data

## Abstract

**Background:**

There is no any large randomized clinical trial of antihypertensive drug treatment with 18-year passive follow-up to examine the risk of Alzheimer’s Disease (AD) or Related Dementias (ADRD).

**Methods:**

Post-trial passive follow-up study of Antihypertensive and Lipid-Lowering Treatment to Prevent Heart Attack Trial (ALLHAT) participants in 1994–1998 by linking with their Medicare claims data through 2017 among 17,158 subjects in 567 U.S. centers who were free of ADRD at baseline on January 1, 1999. Main outcome was the occurrence of ADRD over 18 years of follow-up.

**Results:**

The 18-year cumulative incidence rates were 30.9% for AD, 59.2% for non-AD dementias, and 60.9% for any ADRD. The 18-year cumulative incidence of AD was almost identical for the 3 drug groups (30.5% for chlorthalidone, 31.1% for amlodipine, and 31.4% for lisinopril). The hazard ratios of AD, non-AD dementias and total ADRD were not statistically significantly different among the 3 drug groups. The adjusted hazard ratio of AD was 1.04 (95% CI: 0.94–1.14) for chlorthalidone *versus* amlodipine, 1.02 (0.92–1.13) for lisinopril *versus* amlodipine, and 0.98 (0.89–1.08) for lisinopril *versus* chlorthalidone, which were not significantly different. The risk of AD and non-AD dementias was significantly higher in older subjects, females, blacks, non-Hispanics, subjects with lower education, and subjects with vascular diseases.

**Conclusion:**

The risk of ADRD did not vary significantly by 3 antihypertensive drugs in ALLHAT trial participants with 18-years of follow-up. The risk of ADRD was significantly associated with age, gender, race/ethnicity, education, and history of vascular diseases.

## Introduction

Alzheimer’s Disease (AD) is the most common type of dementia, accounting for an estimated 60%–80% of all dementias, followed by vascular dementia (5%–10%) and dementia with Lewy bodies (5–10%). The prevalence of Alzheimer’s Disease and Related Dementias (ADRD) has been increasing over the past few decades [1–7] and is projected to be doubled by 2025 and tripled by 2050 [1–4]. Causes of ADRD remained largely unknown and have been linked with a number of risk factors, including age, education, social and cognitive engagement, history of psychiatric disorders, head trauma, genetic factors, and vascular diseases or related risk factors (cardiovascular disease, hypertension, diabetes, stroke, and smoking) [1–8]. Antihypertensive drug therapies have been documented to be associated with a lower risk of developing dementia and early cognitive impairment [9–15]. For example, the Systolic Blood Pressure Intervention Trial (SPRINT) Memory and Cognition in Decreased Hypertension (MIND) study reported that intensive Blood Pressure (BP) control significantly reduced the risk of mild cognitive impairment[13,14]. Given the high prevalence of hypertension in the U.S. adult population, it is possible that adequate utilization of disease-modifying drugs may provide an effective strategy for the prevention of cognitive impairment and dementia. Antihypertensive and Lipid-Lowering Treatment to Prevent Heart Attack Trial (ALLHAT), was a large multicenter, randomized, and double-blind trial conducted in 42,418 participants aged ≥ 55 years with hypertension from 1994 to 2002. Data of ALLHAT participants have been linked with their Medicare data to December 2017, encompassing a follow-up to 23 years. This approach enabled us to test the hypothesis if the long-term incidence of developing ADRD from their comprehensive medical records of universal Medicare coverage differed by different antihypertensive drugs used among the trial participants and by other sociodemographic factors or vascular diseases. Furthermore, BP control in the ALLHAT in-trial period and type of different antihypertensive drugs used during the post-trial follow-up period from Medicare Part-D enabled us to address how BP and changes in these drugs affect the outcomes of ADRD. This is the first study to examine the long-term risk of ADRD in association with different types of antihypertensive drugs and BP controls in ALLHAT trial participants, adding new information to the current literature.

## Materials and Methods

### Study population and data sources

The detailed methods of ALLHAT have been reported previously[16].

**Figure 1:**
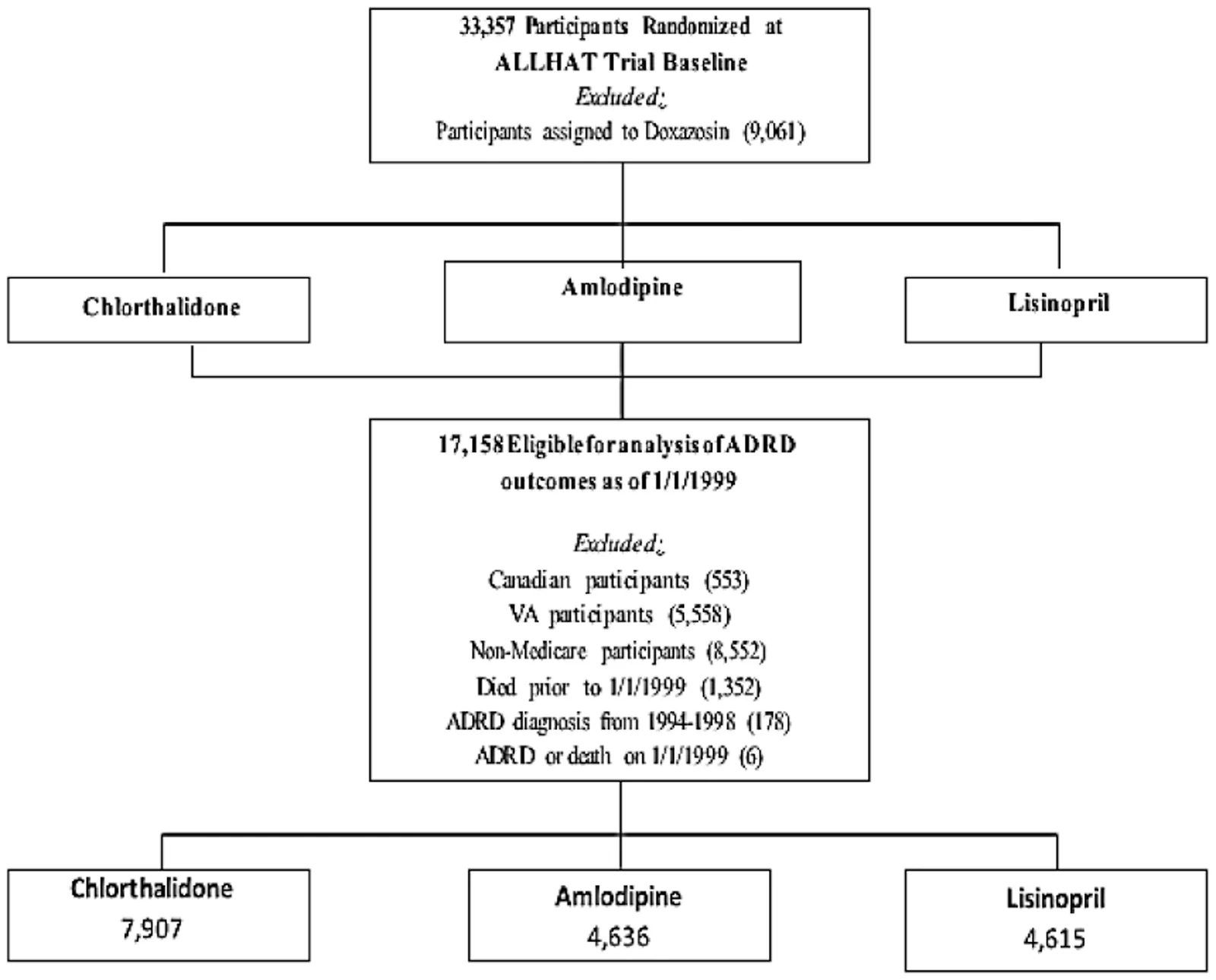
Flowchart of study subjects for inclusion and exclusion.

In brief, ALLHAT was a multicenter, randomized, double-blind, active-controlled trial conducted in 42,418 participants aged ≥ 55 with hypertension and at least one other Coronary Heart Disease (CHD) risk factor in 623 North American centers. Those patients who were eligible and agreed to participate were randomly assigned to 4 treatment arms: an Angiotensin-Converting Enzyme (ACE) inhibitor (lisinopril) (n=9,054), Calcium Channel Blocker (CCB, amlodipine) (n=9,048), α-blocker (doxazosin) (n=9,061), or a thiazide-type diuretic (chlorthalidone) (n=15,255). This study did not include subjects on doxazosin because this arm was terminated earlier. Data for ALLHAT participants aged ≥ 65 have been linked with their Medicare inpatient claims data from January 1, 1994 to December 31, 1998, while their Medicare inpatient, outpatient and physician professional claims data were available from January 1, 1999 to December 31, 2017. Of the 33,357 participants (n=9,054 for lisinopril, n=9,048 for amlodipine, and n=15,255 for chlorthalidone), the study excluded the following subjects (Figure 1): Canadian participants (553), VA participants (5,558), non-Medicare participants (8,552), those who died prior to 1/1/1999 (1,352), or those who had ADRD or died on 1/1/1999 (6). This left 17,158 subjects in final analysis of this study (n=7,907 for chlorthalidone, n=4,636 for amlodipine, and n=4,615 for lisinopril) in 567 U.S. centers who were free of ADRD at baseline on January 1, 1999.

#### Main exposures:

Main exposures for this study were antihypertensive drugs (lisinopril, amlodipine, and chlorthalidone) which were allocated to participants through initial trial randomization and continued on January 1, 1999 as the baseline for this passive follow-up through December 31, 2017. Study exposures also included the BP changes (the latest BP reading prior to 1/1/1999 minus BP at the trial baseline) and post-trial antihypertensive medications based on the 16 major categories of drugs (Supplement Table S1) from Medicare Part-D drug data during 2007–2017.

#### Main outcomes:

Main outcomes were the occurrence of AD, non-AD dementias, and any ADRD combined. AD and non-AD dementias were defined if there were ICD-9 or ICD-10 diagnosis codes for them in Medicare claims data (inpatient, outpatient and physician professional claims) that occurred on one or more occasions after the baseline (January 1, 1999) to the date of last follow-up (December 31, 2017) (Supplement Table S2). The combined ADRD was defined if AD or non-AD dementias were present. For sensitivity analyses, the findings were presented when ADRD was defined from any diagnosis codes and from primary diagnosis code only (i.e., the first diagnosis code out of 12 diagnosis codes) that occurred on at least two separate occasions with 30-days apart in Medicare claims data from 1999 to 2017 (Supplement Figures S1 and S2) (Supplement Tables S3 and S4).

#### Covariates:

ALLHAT baseline (from randomization) demographic and clinical data were incorporated into analyses, including age, gender, race/ethnicity, education, prior receipt of antihypertensive drug therapy, estrogen use (for women), smoking, history of atherosclerotic cardiovascular disease, other atherosclerotic cardiovascular disease, and obesity (body mass index, BMI ≥ 30 kg/m^2^). Wherever possible, covariate data were gathered from extension trial follow-up visits that were most proximal to, but did not extend beyond, 1/1/1999. Extension trial data up to 1/1/1999 were available for aspirin use, High-Density Lipoprotein (HDL) cholesterol level <35 mg/dL, diabetes, history of coronary heart disease, coronary artery bypass graft, major ST segment depression, left ventricular hypertrophy by Minnesota code, and systolic and diastolic blood pressure readings. In the absence of targeted follow-up during the extension phase on the status of Myocardial Infarction (MI) or stroke, data on the history of MI or stroke were supplemented with relevant diagnosis codes garnered from Medicare claims data from January 1, 1994 to December 31, 1998.

### Statistical analysis

Baseline characteristics among the study comparison groups were compared using chi-square statistics for categorical variables and oneway Analysis Of Variance (ANOVA) for continuous variables. The 10-year and 18-year cumulative incidence rates of AD, non-AD dementias, and ADRD were calculated from the baseline on January 1, 1999 to the date of last follow-up (December 31, 2017) using Kaplan-Meier method. The population at risk was those free of dementia at the baseline in 1999. In addition, Cox regression models were used to perform time-to-event analyses to determine the risk of developing ADRD by the 3 study drugs while adjusting for all measured confounding factors listed in tables. The proportionality assumptions for multivariable models was assessed by the Schoenfeld residuals test and by visually inspecting whether the log-log Kaplan-Meier curves are parallel and do not intersect. The Fine and Gray competing risk proportional hazards regression was performed to take death into consideration as a competing risk. In other models, death was considered as a censoring event. Analyses were conducted using R version 4.0.2 (R Foundation for Statistical Computing).

## Results

The comparison of The comparison of baseline characteristics was presented among study participants with 3 antihypertensive drugs (chlorthalidone, amlodipine, and lisinopril) on January 1, 1999. Of 17,158 subjects who were free of ADRD, the baseline characteristics such as age, gender, and race/ethnicity, history of vascular diseases, diabetes and obesity are similar without statistically significant differences among the 3 drug groups. The major ST segment depression was significant at p<0.05 among these 3 groups. Systolic and diastolic BP changes per 10 mmHg from the trial baseline to the latest BP reading prior to 1/1/1999 were significantly different among the 3 groups (p<0.001) (Table 1).

The cumulative incidence of AD, non-AD dementias, and total ADRD that occurred over next 10 and 18 years of follow-up from January 1, 1999 to December 31, 2017 was calculated.. The overall 10-year and 18-year cumulative incidences were 16.3% and 30.9% for AD, 35.4% and 59.2% for non-AD dementias, and 37.5% and 60.9% for any ADRD. The cumulative incidence of AD and non-AD dementias was almost identical for the 3 drug groups (chlorthalidone, amlodipine, and lisinopril), for example, the 30.5%, 31.1% and 31.4% for 18-year cumulative incidence of AD, and 59.0%, 59.9% and 58.6%, respectively for 18-year cumulative incidence of non-AD dementias (Table 2).

The Kaplan-Meier cumulative incidence of AD, non-AD dementias, and ADRD over 18 years of follow-up from 1991 to 2017 by the 3 study drugs, indicating that cumulative incidence curves of AD, non-AD dementias and total ADRD were similar without statistical significance (p>0.05) was shown by the 3 study drugs, (Figure 1).

The 10-year cumulative incidence of AD increased by age from 8.4% for age <70 to 18.2% for age 70–79 and 32.8% for age ≥ 80, whereas the 18-year cumulative incidence of AD increased from 21.7% to 34.5% for age 70–79 and 48.5% for age ≥ 80. The cumulative incidence of ADRD was higher in females, blacks, non-Hispanics, and those with high school or less education than their counterparts. Small variations in cumulative incidences were observed with regards of prior antihypertensive medication use, aspirin use, taking estrogen (for women), cholesterol level, and smoking status. However, those with a history of diabetes, coronary heart disease, atherosclerotic cardiovascular disease, myocardial infarction or stroke, coronary artery bypass graft, and left ventricular hypertrophy by Minnesota Code had a higher cumulative incidence of ADRD (Figures 2a–2c).

The time to event unadjusted and adjusted hazard ratios of developing ADRD. The hazard ratios of AD, non-AD dementias and total ADRD were not statistically significantly different among the 3 drug groups. For example, the hazard ratio of AD was 1.04 (95% CI: 0.94–1.14) for chlorthalidone *versus* amlodipine, 1.02 (0.92–1.13) for lisinopril *versus* amlodipine, and 0.98 (0.89–1.08) for lisinopril *versus* chlorthalidone, after adjusting for other confounding factors. However, the adjusted risk of AD and non-AD dementias was significantly higher in subjects aged 70–79 (2.00, 1.81–2.20 for AD and 1.65, 1.55–1.75 for non-AD dementias) and in those aged ≥ 80 (3.88, 3.43–4.39 and 3.45, 3.18–3.74) as compared to those aged <70. The adjusted risk of AD was 13% higher (1.13, 1.03–1.24) and the risk of non-AD dementias was 9% higher (1.09, 1.06–1.16) in females as compared to males. The risk of AD and non-AD dementias was significantly higher in blacks and non-Hispanics. Subjects with lower education (high school or lower) appeared to have a significantly higher adjusted risk of AD (1.11, 1.01–1.22), non-AD dementias (1.13, 1.07–1.21), and any ADRD (1.12, 1.05–1.19) as compared to those with higher education. Those who received antihypertensive drugs or aspirin prior to the start of study and those with lower cholesterol did not have a significantly increased risk of AD or non-AD dementias than their counterparts. Women taking estrogen at baseline had a significantly lower risk of AD and any ADRD. Those current smokers but not former smokers had a significantly higher risk of non-AD dementias and ADRD. Subjects with a history of diabetes had a significantly higher adjusted risk of AD, non-AD dementias and any ADRD, whereas those with atherosclerotic cardiovascular disease had a significantly higher risk of AD and any ADRD combined but had an insignificant risk of non-AD dementias. Subjects with myocardial infarction or stroke and those with left ventricular hypertrophy by Minnesota code had a significantly higher risk of non-AD dementias and any ADRD but had an insignificant risk of AD. Subjects with coronary heart disease, coronary artery bypass graft, major ST segment depression, or participation in the Lipid Lowering Trial did not have a significantly different risk of AD, non-AD dementias and any ADRD. Those with obesity had a significantly lower risk of AD but had no significantly different risk of non-AD dementia or any ADRD as compared to those without obesity. Table 3 also shows that a reduction in systolic and diastolic BP per 10 mmHg from the trial baseline to the latest BP prior to 1999 was not associated with a significantly different risk of AD and non-AD dementias after adjusting for other factors (Table 3).

The risk of developing ADRD were analyzed. In association with the 3 study drugs, sociodemographic factors, and vascular diseases was presented by taking into consideration death before ADRD as a competing risk in the Cox regression models in addition to adjusting for multiple confounders. The results on the risk of AD, non-AD dementias, and any ADRD by the 3 study drugs, age, gender, race/ethnicity, education, and smoking were generally similar to what were presented in Table 3, although the magnitude of hazard ratios changed slightly (Table 4).

In sensitivity analyses, using a stricter definition of ADRD that occurred at least on 2 occasions with 30 days apart in Medicare claims data, the Kaplan-Meier cumulative incidence rates of AD, non-AD dementias, and any ADRD by the 3 study drugs over the 18 years of follow-up are shown in supplemental Figure-S1 from any diagnosis codes and in Figure S2 from primary diagnosis codes only. The overall 18-year cumulative incidence of AD, non-AD dementias, and any ADRD was 19.9%, 42.0%, and 44.1%, respectively, from any diagnosis codes, and 10.3%, 23.7%, and 27.6%, respectively, from primary diagnosis codes only. There were no statistically significant differences in cumulative incidences by the 3 study drugs. The overall patterns in the adjusted risks of AD, non-AD dementias and any ADRD in association with the 3 study drugs, age, gender, race/ethnicity, education, and history of vascular diseases, which were presented in supplemental (Tables S3 and S4), were similar to those presented in Tables 3 and 4. In addition, we examined the effects of post-trial antihypertensive drug uses on the risk of ADRD among 6086 subjects who were enrolled in Medicare Part-D drug program in 2007–2017, stratified by 3 study drugs (Table S5). As compared to those who received the 3 study drugs (ACE, lisinopril; CCB, amlodipine; and thiazide-type diuretic, chlorthalidone), subjects who continued to receive it and those who did not continue it but received 1 to 4 other categories of antihypertensive drugs did not have significantly different risks of ADRD.

## Discussion

This study examined a large number of subjects who participated in multi-center phase-3 trial of antihypertensive drugs with up to 18 years of passive follow-up to determine the risk of AD, non-AD dementias and total ADRD from 1999 to 2017. This was the first report of this large ALLHAT multicenter clinical trial on the association between 3 anti-hypertensive drugs and the risk of ADRD with up to 18 years of follow-up, contributing significantly to the existing knowledge. The study found that subjects who received 3 different antihypertensive drugs and who were free of dementia on January 1, 1999 did not have significantly different risks of AD, non-AD dementias, and total ADRD over the next 18 years of follow-up. The risk of AD and non-AD dementias significantly increased with age and was significantly higher in females, blacks, non-Hispanics, and those with lower education. Those with a history of diabetes and cardiovascular diseases had a significantly higher risk of AD and non-AD dementias. The reduction in both systolic and diastolic BP from the trial baseline to the latest BP prior to 1999 was not associated with a significantly different risk of AD and non-AD dementias after adjusting for confounders. Furthermore, post-trial antihypertensive drug uses from Medicare Part-D drug files were found to have no significant impact on the risk of ADRD.

Numerous studies demonstrated that hypertension is associated with an increased risk of late cognitive decline and ADRD [9–15], Previous studies on the mechanism of the relationship between hypertension and ADRD indicated that hypertension may alter cerebrovascular autoregulation, increase susceptibility to ischemia, impede flow and damage the blood–brain barrier via vascular narrowing, vascular stiffness, and endothelial dysfunction, and induce oxidative stress that may increase the risk of dementia [17–18]. Antihypertensive treatment and BP lowering were expected to be associated with a reduction in the risk of ADRD. However, many studies, including some randomized clinical trials, produced mixed results about the impact of hypertension treatment on the risk of ADRD. For example, Honolulu-Asia Aging Study and the Rotterdam study found that hypertension with treatment was associated with a decreased incidence of poor cognitive function and dementia, whereas the Framingham study and the Washington Heights study found no significant relationships between hypertension or BP reduction and cognitive function or risk of dementia [19–22]. Several clinical trials, including Hypertension in the Very Elderly (HYVET) Trial and Systolic Hypertension in the Elderly Project (SHEP) did not find a significant effect of hypertension treatment on the reduced risk of dementia[18,23,24]. A clinical trial in the elderly patients with hypertension found that higher pulse pressures were associated with a higher risk of dementia and diastolic BP showed a J-shape relationship with the risk of dementia [25]. However, a more recent large Systolic Blood Pressure Intervention Trial (SPRINT) found that antihypertensive drug therapies were associated with a significantly lower risk of developing mild cognitive impairment [13,14]. This trial reported that intensive BP control significantly reduced the risk of mild cognitive impairment (with an incidence of 14.6 and 18.3 cases per 1000 person-years between 2 treatment arms and hazard ratio of 0.81; 95% CI of 0.69–0.95) [13]. The study was conducted in 9,361 patients aged ≥ 50 with hypertension, in whom mean age was 67.9 years and 28.2% of participants were ≥ 75 years. The mechanism for an effective strategy for prevention of cognitive impairment may be related to amyloid-β production, neurovascular coupling and cerebral perfusion, and clearance of free radical species as documented in animal studies[13,14]. However, this trial did not address the effects of specific antihypertensive drugs on the risk of cognitive impairments and ADRD. Recently, a meta-analysis of 6 prospective community-based studies concluded that antihypertensive treatment was associated with a significantly reduced risk of AD (hazard ratio: 0.84, 95% CI: 0.73–0.97) and overall dementias (0.88, 0.79–0.98), but did not find significant differences between one drug class *versus* all others on the risk of dementia [27–31]. Although some studies demonstrated that because of potential neuroprotective effects, calcium channel blockers such as amlodipine were associated with a decreased risk of dementia, other studies showed that calcium channel blockers were not significantly associated with a risk of dementia [32–34]. For example, a systematic review of calcium channel blocker use and cognitive decline/dementia in the elderly, including cohort studies and clinical trials, concluded that there was no clear evidence to suggest that calcium channel blockers was associated with the risk of cognitive decline or dementia in the very elderly [34]. It is possible that calcium channel blockers are associated with a decreased risk of incident dementia in a younger population with hypertension. However, our study using Medicare data in the elderly beneficiaries cannot address this research question in younger population.

Because ADRD takes a long time to develop, is relatively rare in young population, and is uncommon at the early stage of study follow-up, a very large number of study population and a long-term follow-up are often required in order to generate meaningful information on the incidence of ADRD. Previous clinical trials on hypertension and dementia had a relatively small number of participants, short follow-up time, and assessment of mild cognitive impairment rather than late stage dementia. ALLHAT trial had a large number of participants (mean age at enrollment was 67) with hypertension that started in 1994 and completed in 2002. Importantly, the data of ALLHAT trial participants were linked with their Medicare claims data through December 2017 (mean age was 73 at baseline of 1999), making the study unique to determine the long-term risk of ADRD. These nationwide Medicare claims data are the comprehensive medical records for their beneficiaries from the time of enrollment to the time of death regardless of where they seek medical care across the country, hence providing a long and most comprehensive history of medical records to capture the incidence of dementias. The study found a number of common risk factors for AD and ADRD, including age and history of vascular diseases, but also found that there were no significantly different effects of 3 hypertensive drugs on the risk of ADRD. However, the relationship between hypertension and ADRD are complex. Some studies explored the complex relationship between hypertension and ADRD, and found that hypertension at midlife is a risk factor for ADRD but hypertension at late life may have no effect or a weak protective effect for ADRD [35,36].

Our study has some limitations. First, we included ALLHAT participants who were still alive at baseline in 1999 and enrolled in Medicare program. Hence, initial trial randomization was no longer intact and any analyses done off-randomization may be subject to unmeasured or unknown confounders. Although the distribution of all baseline characteristics among three study drug groups after excluding some subjects from the original samples was not significantly different and we adjusted for measured confounders, unmeasured or unknown confounders among the comparison groups may still be present, affecting the study findings. Second, BP measures were not available from Medicare claims during the post-trial period; hence it was not known how well BP was controlled. Even though we controlled for BP changes from the trial baseline to the latest BP prior to 1999, BP levels in more recent years should be helpful to assess the effect of BP levels on the risk of ADRD. Third, the study excluded those trial participants who were from Canada and Veteran Affairs (VA) because of their being ineligible for Medicare or having incomplete Medicare claims, the findings may just be generalizable to those Medicare beneficiaries in the U.S. Fourth, Medicare claims were reported to have a sensitivity of >85% for ADRD and 79% for AD, and specificity of 89% for ADRD [37]. Hence, there could be a certain degree of underestimation, overestimation, or misclassification of types of ADRD diagnoses based on Medicare claims data. Furthermore, study cannot determine if a higher risk of ADRD in those with lower education was due to late access to medical care for diagnosis or if it was due to other protective factors related to higher education. Finally, although the effects of post-trial antihypertensive medications on the risk of ADRD was explored in sensitivity analyses, it was only examined in those enrolled in Medicare Part-D drug program in 2007–2017, but not in an entire study population [38].

## Conclusion

In conclusion, the study found that, in a large number of subjects who participated in multicenter phase-3 trial of antihypertensive drugs with up to 18 years of passive follow-up, subjects with 3 different antihypertensive drugs did not have significantly different risks of AD, non-AD dementias, and total ADRD. The risk of AD and non-AD dementias was significantly associated with age, gender, race/ethnicity, and education. Those with a history of diabetes, atherosclerotic cardiovascular disease, myocardial infarction or stroke, and left ventricular hypertrophy by Minnesota code had a significantly higher risk of AD and non-AD dementias. The reduction in systolic and diastolic BP from the trial baseline to the latest BP prior to 1999 was not associated with a significantly different risk of AD or non-AD dementias. The receipt of post-trial antihypertensive drugs in 2007–2017 did not significantly affect the risk of dementias. Future research may be helpful to examine the risk of specific type of dementias, including AD, vascular dementia, dementia with Lewy bodies, Frontotemporal degeneration and dementias, and mild cognitive impairment, in association with the use of these different antihypertensive drugs.

## Supplementary Material

Supplement File

## Figures and Tables

**Figure 2a:**
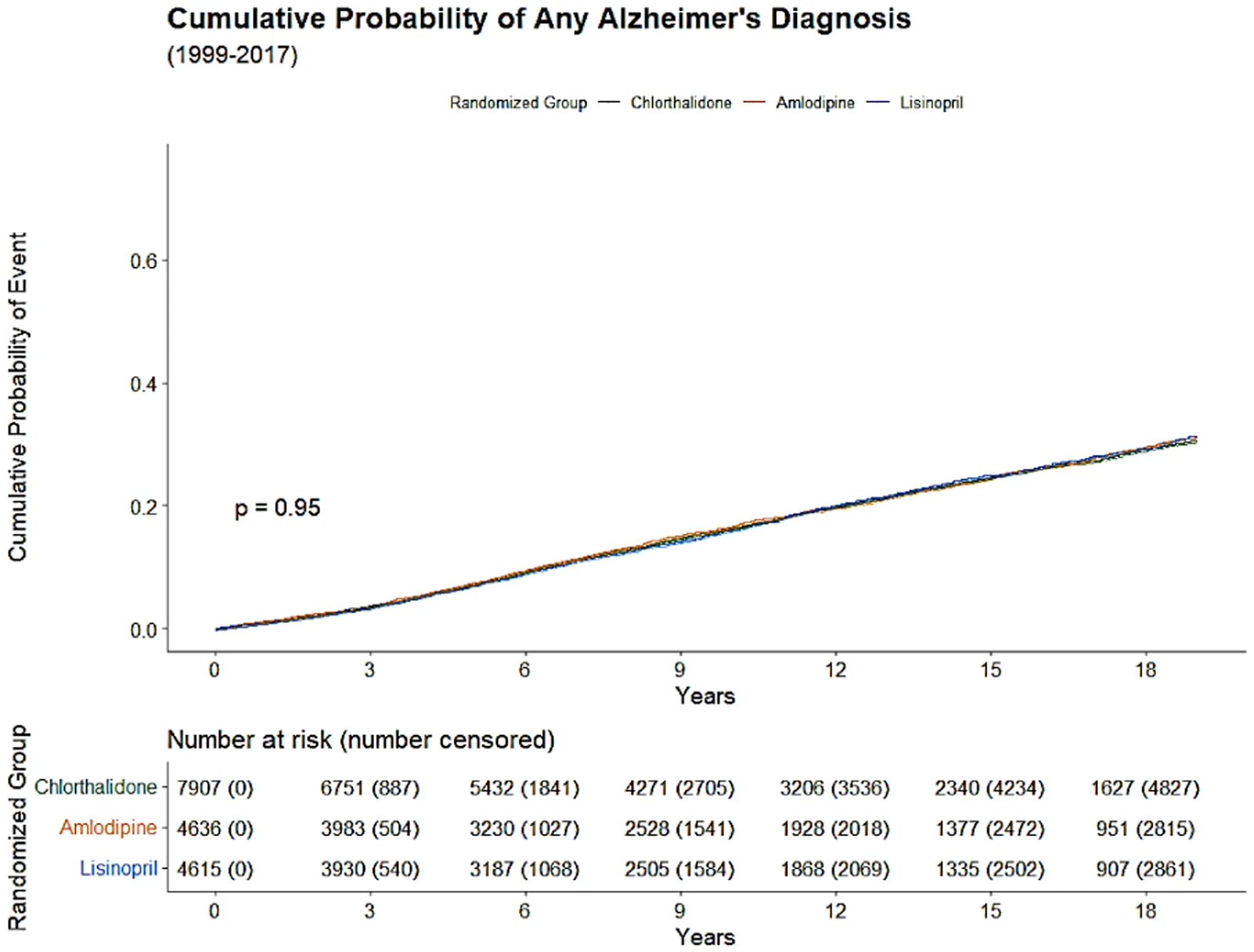
Kaplan-Meier cumulative incidence of Alzheimer’s Disease (AD) from 1999 to 2017.

**Figure 2b:**
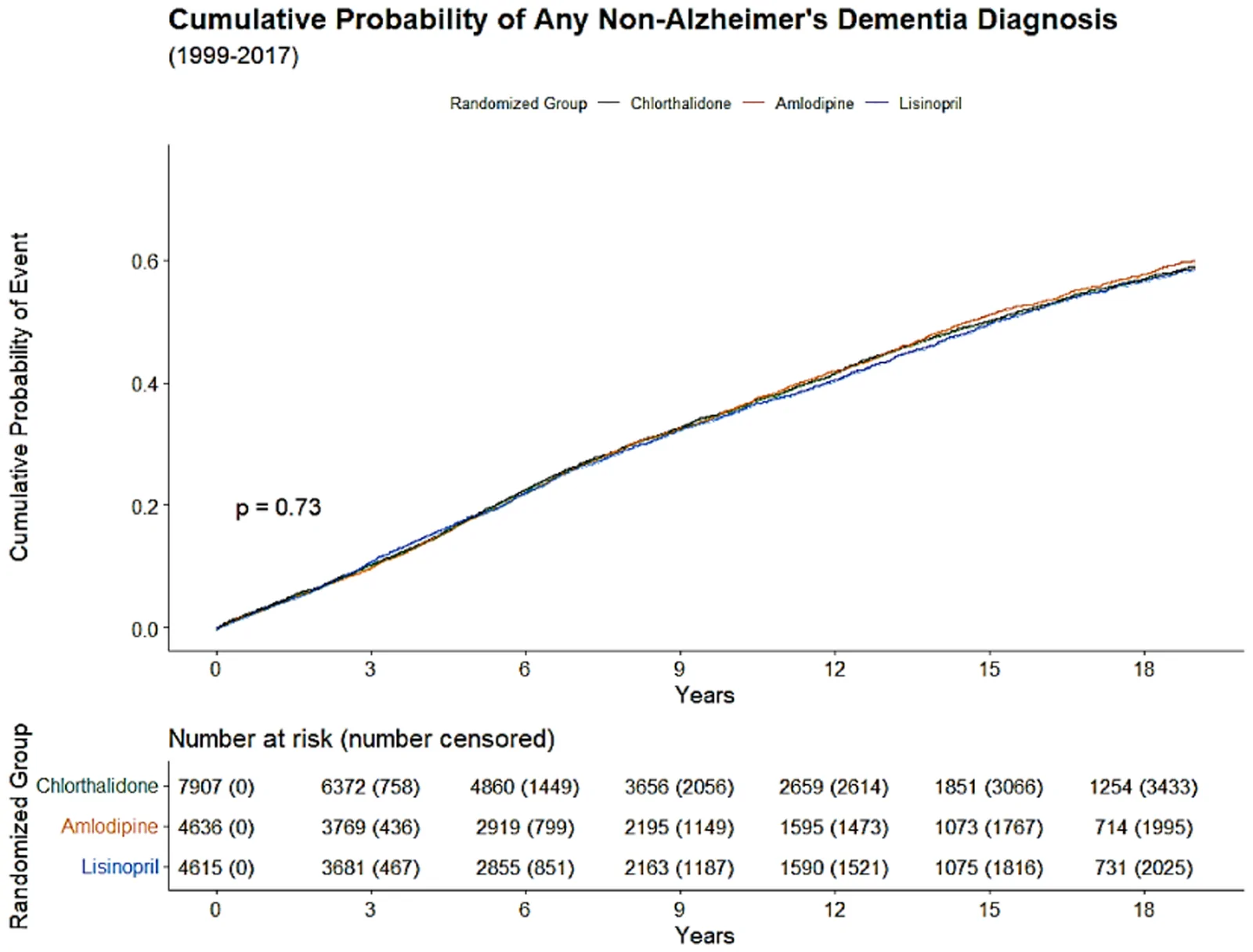
Kaplan-Meier cumulative incidence of non-AD Dementias (2b), from 1999 to 2017.

**Figure 2c:**
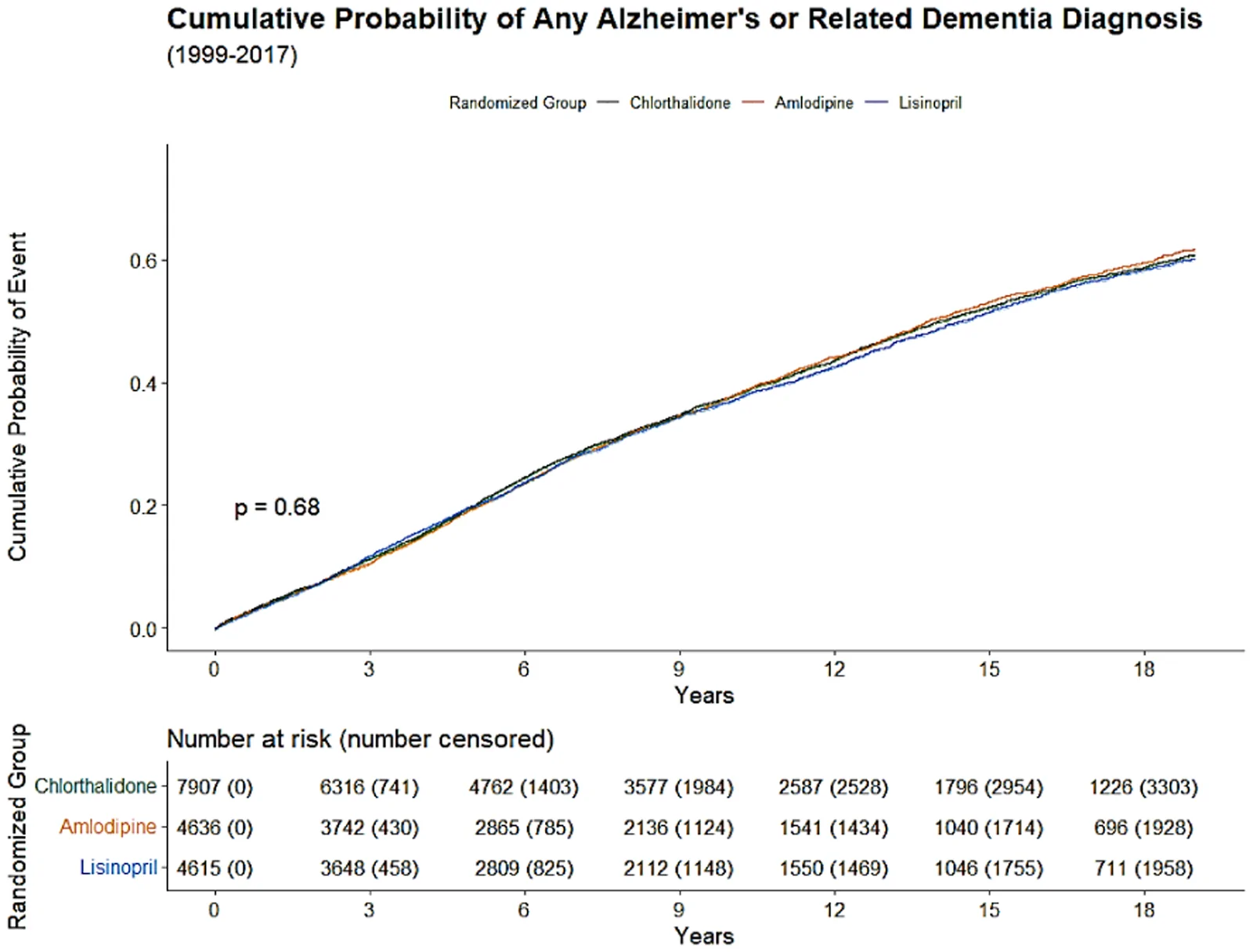
Kaplan-Meier cumulative incidence of any ADRD (2c) from 1999 to 2017.

**Table 1:** Baseline characteristics by the 3 study drugs in allhat participants.

S.No	Full Sample	Chlorthalidone	Amlodipine	Lisinopril	P value*^†^
**Participants, N (%) (Unless otherwise indicated)**					
Eligible for further follow-up as of 1/1/1999	17158	7907	4636	4615	NA
Age, mean (SD), years	73.38 (6.31)	73.31 (6.26)	73.44 (6.37)	73.44 (6.34)	0.384
**Age group (as of 1/1/1999)**					
Age <70	6016 (35.1)	2815 (35.6)	1602 (34.6)	1599 (34.6)	0.445
Age 70–79	8403 (49.0)	3871 (49.0)	2272 (49.0)	2260 (49.0)	
Age 80+	2739 (16.0)	1221 (15.4)	762 (16.4)	756 (16.4)	
**Gender**					
Male	7366 (42.9)	3392 (42.9)	1945 (42.0)	2029 (44.0)	0.148
Female	9792 (57.1)	4515 (57.1)	2691 (58.0)	2586 (56.0)	
**Race/Ethnicity**					
Black	5942 (34.6)	2735 (34.6)	1608 (34.7)	1599 (34.6)	0.994
Non-Black	11216 (65.4)	5172 (65.4)	3028 (65.3)	3016 (65.4)	
**Hispanic/Latino Ethnicity**					
Hispanic	3618 (21.2)	1661 (21.1)	954 (20.7)	1003 (21.9)	0.376
Non-Hispanic	13453 (78.8)	6209 (78.9)	3658 (79.3)	3586 (78.1)	
**Education level**					
Education, mean (SD), years	10.58 (4.21)	10.60 (4.19)	10.55 (4.13)	10.57 (4.33)	0.781
High school or less	11743 (74.0)	5391 (73.6)	3192 (74.4)	3160 (74.2)	0.578
More than high school	4123 (26.0)	1931 (26.4)	1096 (25.6)	1096 (25.8)	
**Treatment with antihypertensive drugs prior to trial baseline**					
Treated	15554 (90.7)	7137 (90.3)	4218 (91.0)	4199 (91.0)	0.269
Untreated	1604 (9.3)	770 (9.7)	418 (9.0)	416 (9.0)	
Aspirin use (as of 1/1/1999)	6347 (37.3)	2924 (37.4)	1689 (36.7)	1734 (37.9)	0.497
Women taking estrogen at trial baseline	1433 (14.9)	692 (15.6)	381 (14.4)	360 (14.2)	0.18
HDL cholesterol (as of 1/1/1999), mean (SD), mg/dl	48.13 (14.82)	48.05 (14.83)	48.53 (15.03)	47.85 (14.57)	0.079
HDL <35 mg/dl (as of 1/1/1999)	2764 (16.1)	1279 (16.2)	710 (15.3)	775 (16.8)	0.151
**Cigarette smoking at trial baseline**					
Never smoker	7562 (44.1)	3460 (43.8)	2070 (44.7)	2032 (44.0)	0.296
Current Smoker	2811 (16.4)	1315 (16.6)	777 (16.8)	719 (15.6)	
Former Smoker	6784 (39.5)	3132 (39.6)	1789 (38.6)	1863 (40.4)	
**Diabetes classification (as of 1/1/1999)**					
Diabetes	7012 (44.1)	3251 (44.3)	1898 (44.2)	1863 (43.5)	0.707
Non-diabetes	8900 (55.9)	4091 (55.7)	2392 (55.8)	2417 (56.5)	
History of Coronary Heart Disease (CHD) (as of 1/1/1999)	4792 (27.9)	2246 (28.4)	1251 (27.0)	1295 (28.1)	0.225
Atherosclerotic Cardiovascular Disease (ASCVD) at trial baseline	9375 (54.6)	4374 (55.3)	2465 (53.2)	2536 (55.0)	0.058
History of Myocardial Infarction (MI) or stroke (as of 1/1/1999)	4606 (26.8)	2124 (26.9)	1254 (27.0)	1228 (26.6)	0.891
History of Coronary Artery Bypass Graft (CABG) (as of 1/1/1999)	2652 (15.5)	1233 (15.6)	675 (14.6)	744 (16.1)	0.104
Other ASCVD at trial baseline	4516 (26.3)	2084 (26.4)	1205 (26.0)	1227 (26.6)	0.806
Major ST segment depression (as of 1/1/1999)	1374 (8.0)	662 (8.4)	331 (7.2)	381 (8.3)	0.037
Left Ventricular Hypertrophy (LVH) by Minnesota code (as of 1/1/1999)	658 (4.3)	307 (4.3)	178 (4.3)	173 (4.2)	0.95
Lipid Lowering Trial (LLT) Participants	4181 (24.4)	1961 (24.8)	1140 (24.6)	1080 (23.4)	0.195
Body Mass Index (BMI), mean (SD), kg/m^2^ at trial baseline	29.40 (5.98)	29.35 (6.03)	29.44 (5.98)	29.45 (5.92)	0.609
Obesity (BMI ≥30 kg/m^2^) at trial baseline	6821 (39.8)	3125 (39.5)	1847 (39.8)	1849 (40.1)	0.828
**Latest Blood Pressure (BP) reading prior to 1/1/1999, mmHg**					
Systolic BP, mean (SD)	140.10 (16.58)	139.23 (16.23)	140.12 (15.72)	141.56 (17.85)	<0.001
Diastolic BP, mean (SD)	78.93 (10.03)	79.04 (9.90)	78.33 (9.87)	79.34 (10.40)	<0.001
**Blood pressure change (the latest BP reading prior to 1/1/1999 minus BP at trial baseline), mmHg**					
Systolic BP, mean (SD)	−7.19 (18.35)	−8.08 (17.90)	−6.92 (18.26)	−5.95 (19.13)	<0.001
Diastolic BP, mean (SD)	−4.41 (10.54)	−4.50 (10.44)	−4.82 (10.50)	−3.85 (10.73)	<0.001

**Abbreviations:** N: Number Of Participants; ADRD: Alzheimer’s Disease and Related Dementias; SD: Standard Deviation.

Note:

* P values represent significance level of the chi-square test of independence between randomized groups for binary and categorical variables, or the one-way Analysis Of Variance (ANOVA) between randomized groups for continuous variables,

† Statistically significant p values (<0.05) are shown in bold text.

**Table 2:** Cumulative incidence (%) of adrd by randomized group (3 study drugs) and other factors (1999 to 2017).

Demographic	Alzheimer’s Disease (AD)			Other non-AD Dementias			All ADRD combined		
	Events/Total (n/N)	10-Year Rate/100 Persons (SE)	18-Year Rate/100 Persons (SE)^†^	Events/Total	10-Year Rate/100 Persons (SE)	18-Year Rate/100 Persons (SE)^†^	Events/Total	10-Year Rate/100 Persons (SE)	18-Year Rate/100 Persons (SE)^†^
All Patients	3247/17158	16.3 (0.3)	30.9 (0.5)	7127/17158	35.4 (0.4)	59.2 (0.5)	7461/17158	37.5 (0.4)	60.9 (0.5)
**Randomized group**									
Chlorthalidone	1485/7907	16.3 (0.5)	30.5 (0.7)	3277/7907	35.6 (0.6)	59.0 (0.7)	3438/7907	37.8 (0.6)	60.9 (0.7)
Amlodipine	890/4636	16.6 (0.6)	31.1 (1.0)	1960/4636	35.7 (0.8)	59.9 (1.0)	2046/4636	37.7 (0.8)	61.7 (1.0)
Lisinopril	872/4615	16.0 (0.6)	31.4 (1.0)	1890/4615	35.0 (0.8)	58.6 (1.0)	1977/4615	37.0 (0.8)	60.3 (1.0)
**Age group (as of 1/1/1999)**									
Age <70	843/6016	8.4 (0.4)	21.7 (0.7)	2114/6016	23.6 (0.6)	48.1 (0.8)	2208/6016	25.2 (0.6)	49.8 (0.8)
Age 70–79	1738/8403	18.2 (0.5)	34.5 (0.8)	3627/8403	37.1 (0.6)	63.0 (0.7)	3796/8403	39.4 (0.6)	64.8 (0.7)
Age 80+	666/2739	32.8 (1.2)	48.5 (1.9)	1386/2739	61.7 (1.2)	77.4 (1.3)	1457/2739	64.2 (1.2)	79.5 (1.3)
**Gender**									
Male	1154/7366	14.4 (0.5)	27.4 (0.8)	2749/7366	33.2 (0.6)	56.5 (0.8)	2870/7366	35.0 (0.6)	57.9 (0.8)
Female	2093/9792	17.7 (0.4)	33.2 (0.7)	4378/9792	37.0 (0.5)	60.9 (0.6)	4591/9792	39.3 (0.5)	63.0 (0.6)
**Race/ethnicity**									
Black	1228/5942	17.8 (0.6)	33.8 (0.9)	2667/5942	39.1 (0.7)	63.6 (0.8)	2772/5942	40.8 (0.7)	65.1 (0.8)
Non-Black	2019/11216	15.5 (0.4)	29.4 (0.6)	4460/11216	33.6 (0.5)	56.8 (0.6)	4689/11216	35.8 (0.5)	58.7 (0.6)
**Hispanic/Latino Ethnicity**									
Hispanic	689/3618	17.7 (0.7)	26.2 (0.9)	983/3618	24.1 (0.8)	36.9 (1.0)	1188/3618	30.2 (0.8)	42.8 (1.0)
Non-Hispanic	2542/13453	16.0 (0.4)	32.6 (0.6)	6104/13453	38.5 (0.5)	65.2 (0.6)	6233/13453	39.6 (0.5)	66.0 (0.6)
**Education level**									
High school or less	2273/11743	17.5 (0.4)	31.7 (0.6)	4900/11743	36.6 (0.5)	59.7 (0.6)	5151/11743	38.9 (0.5)	61.8 (0.6)
More than high school	728/4123	12.9 (0.6)	28.9 (1.0)	1708/4123	32.0 (0.8)	58.1 (1.0)	1764/4123	33.5 (0.8)	59.2 (1.0)
**Treatment with antihypertensive drugs prior to trial baseline**									
Treated	2933/15554	16.3 (0.3)	30.9 (0.5)	6476/15554	35.6 (0.4)	59.5 (0.5)	6778/15554	37.7 (0.4)	61.3 (0.5)
Untreated	314/1604	16.4 (1.0)	31.1 (1.6)	651/1604	34.1 (1.3)	56.0 (1.6)	683/1604	35.6 (1.3)	57.8 (1.6)
**Aspirin use (as of 1/1/1999)**									
Yes	1133/6347	14.9 (0.5)	31.1 (0.9)	2650/6347	34.7 (0.7)	61.5 (0.9)	2752/6347	36.4 (0.7)	63.0 (0.8)
No	2079/10647	17.1 (0.4)	30.8 (0.6)	4416/10647	35.9 (0.5)	57.9 (0.6)	4644/10647	38.2 (0.5)	59.8 (0.6)
**Women taking estrogen at trial baseline**									
Yes	301/1433	15.1 (1.0)	29.7 (1.5)	678/1433	33.9 (1.3)	60.3 (1.6)	697/1433	35.9 (1.3)	61.2 (1.6)
No	1754/8183	18.2 (0.5)	33.8 (0.7)	3629/8183	37.6 (0.6)	61.1 (0.7)	3817/8183	40.0 (0.6)	63.3 (0.7)
**HDL cholesterol <35 mg/dl (as of 1/1/1999)**									
Yes	457/2764	13.9 (0.8)	29.9 (1.4)	1029/2764	32.4 (1.0)	56.7 (1.3)	1080/2764	34.4 (1.0)	58.2 (1.3)
No	2790/14394	16.7 (0.4)	31.1 (0.5)	6098/14394	36.0 (0.4)	59.6 (0.5)	6381/14394	38.1 (0.4)	61.4 (0.5)
**Cigarette smoking at trial baseline**									
Never smoker	1650/7562	18.0 (0.5)	32.7 (0.7)	3372/7562	36.3 (0.6)	59.7 (0.7)	3539/7562	38.7 (0.6)	61.7 (0.7)
Current smoker	422/2811	14.1 (0.8)	29.7 (1.4)	1017/2811	35.0 (1.1)	57.5 (1.4)	1068/2811	36.8 (1.1)	59.7 (1.4)
Former Smoker	1175/6784	15.2 (0.5)	29.3 (0.8)	2737/6784	34.7 (0.7)	59.1 (0.8)	2853/6784	36.5 (0.7)	60.5 (0.8)
**Diabetes classification (as of 1/1/1999)**									
Diabetes	1317/7012	17.1 (0.5)	33.2 (0.9)	2963/7012	38.5 (0.7)	63.1 (0.8)	3109/7012	40.7 (0.7)	65.0 (0.8)
Non-diabetes	1689/8900	15.5 (0.4)	29.4 (0.7)	3633/8900	32.8 (0.5)	56.3 (0.7)	3798/8900	34.9 (0.6)	58.0 (0.7)
**History of Coronary Heart Disease (CHD) (as of 1/1/1999)**									
Yes	842/4792	16.3 (0.6)	31.4 (1.0)	1966/4792	36.6 (0.8)	61.2 (1.0)	2053/4792	38.7 (0.8)	63.0 (1.0)
No	2405/12366	16.3 (0.4)	30.8 (0.6)	5161/12366	35.0 (0.5)	58.4 (0.6)	5408/12366	37.1 (0.5)	60.2 (0.6)
**Atherosclerotic Cardiovascular Disease (ASCVD) at trial baseline**									
Yes	1795/9375	17.4 (0.5)	31.9 (0.7)	4015/9375	37.8 (0.6)	61.7 (0.7)	4184/9375	39.7 (0.6)	63.2 (0.7)
No	1452/7783	15.1 (0.5)	29.7 (0.7)	3112/7783	32.7 (0.6)	56.3 (0.7)	3277/7783	35.0 (0.6)	58.3 (0.7)
**History of Myocardial Infarction (MI) or stroke (as of 1/1/1999)**									
Yes	825/4606	18.5 (0.7)	32.6 (1.1)	1985/4606	42.2 (0.8)	65.3 (1.0)	2059/4606	44.1 (0.8)	66.6 (1.0)
No	2422/12552	15.6 (0.4)	30.3 (0.6)	5142/12552	33.2 (0.5)	57.2 (0.6)	5402/12552	35.3 (0.5)	59.1 (0.6)
**History of Coronary Artery Bypass Graft (CABG) (as of 1/1/1999)**									
Yes	442/2652	15.2 (0.8)	32.6 (1.5)	1100/2652	38.0 (1.1)	64.6 (1.4)	1131/2652	39.3 (1.1)	65.7 (1.4)
No	2805/14506	16.5 (0.4)	30.8 (0.5)	6027/14506	35.0 (0.4)	58.3 (0.5)	6330/14506	37.2 (0.4)	60.2 (0.5)
**Other ASCVD at trial baseline**									
Yes	839/4516	17.0 (0.6)	31.1 (1.0)	1950/4516	37.9 (0.8)	62.0 (1.0)	2023/4516	39.7 (0.8)	63.4 (1.0)
No	2408/12642	16.1 (0.4)	30.8 (0.6)	5177/12642	34.6 (0.5)	58.2 (0.6)	5438/12642	36.8 (0.5)	60.1 (0.6)
**Major ST segment depression (as of 1/1/1999)**									
Yes	241/1374	15.3 (1.1)	29.8 (1.8)	553/1374	36.5 (1.5)	58.1 (1.8)	576/1374	38.2 (1.5)	59.7 (1.8)
No	2996/15732	16.4 (0.3)	31.0 (0.5)	6550/15732	35.4 (0.4)	59.2 (0.5)	6861/15732	37.5 (0.4)	61.0 (0.5)
**Left Ventricular Hypertrophy (LVH) by Minnesota code (as of 1/1/1999)**									
Hard LVH	115/658	19.2 (1.9)	33.3 (3.1)	281/658	46.7 (2.3)	63.2 (2.8)	295/658	48.5 (2.3)	65.1 (2.7)
No/Soft LVH	2770/14632	16.0 (0.3)	30.7 (0.5)	6151/14632	35.2 (0.4)	59.7 (0.5)	6421/14632	37.1 (0.4)	61.3 (0.5)
**Lipid Lowering Trial (LLT) participant**									
Yes	775/4181	15.7 (0.6)	28.6 (1.0)	1699/4181	33.1 (0.8)	56.7 (1.0)	1789/4181	35.5 (0.8)	58.8 (1.0)
No	2472/12977	16.5 (0.4)	31.7 (0.6)	5428/12977	36.2 (0.5)	60.0 (0.6)	5672/12977	38.2 (0.5)	61.6 (0.6)
**Obesity (BMI ≥ 30 kg/m2) at trial baseline**									
Yes	1193/6821	13.7 (0.5)	29.7 (0.8)	2855/6821	34.0 (0.6)	60.3 (0.8)	2954/6821	35.5 (0.6)	61.6 (0.8)
No	2054/10337	18.1 (0.4)	31.7 (0.6)	4272/10337	36.4 (0.5)	58.4 (0.7)	4507/10337	38.9 (0.5)	60.4 (0.6)

Abbreviations: ADRD:Alzheimer’s Disease and Related Dementias; SE:Standard Error

Note:

† 18-year time to event includes events occurring up to, but not including year 19 (e.g. throughout the year 2017)

**Table 3:** Hazard ratio (HR, 95% CI) of ADRD by randomized group (3 study drugs) and other factors (1999 to 2017).

Demographic	Alzheimer’s Disease (AD)				Other non-AD Dementias			All ADRD combined				
	Unadjusted HR (95% CI)	P value	Adjusted* HR (95% CI)	P value	Unadjusted HR (95% CI)	P value	Adjusted* HR (95% CI)	P value	Unadjusted HR (95% CI)	P value	Adjusted* HR (95% CI)	P value
**Randomized group**												
Chlorthalidone vs Amlodipine	1.01 (0.93, 1.10)	0.753	1.04 (0.94, 1.14)	0.434	1.01 (0.95, 1.07)	0.763	1.00 (0.94, 1.06)	0.985	1.01 (0.95, 1.06)	0.858	1.00 (0.94, 1.06)	0.983
Lisinopril vs Amlodipine	1.00 (0.91, 1.10)	0.93	1.02 (0.92, 1.13)	0.733	1.03 (0.96, 1.09)	0.431	0.99 (0.92, 1.06)	0.774	1.03 (0.96, 1.09)	0.416	0.98 (0.92, 1.06)	0.666
Lisinopril vs Chlorthalidone^‡^	0.99 (0.91, 1.08)	0.835	0.98 (0.89, 1.08)	0.694	1.02 (0.96, 1.08)	0.566	0.99 (0.93, 1.06)	0.763	1.02 (0.97, 1.08)	0.47	0.98 (0.92, 1.05)	0.615
**Age group (as of 1/1/1999)**												
Age <70	1.00 (reference)		1.00 (reference)		1.00 (reference)		1.00 (reference)		1.00 (reference)		1.00 (reference)	
Age 70–79	1.91 (1.76, 2.08)	<0.001	2.00 (1.81, 2.20)	<0.001	1.62 (1.54, 1.71)	<0.001	1.65 (1.55, 1.75)	<0.001	1.62 (1.54, 1.71)	<0.001	1.65 (1.55, 1.75)	<0.001
Age 80+	3.87 (3.49, 4.31)	<0.001	3.88 (3.43, 4.39)	<0.001	3.32 (3.09, 3.56)	<0.001	3.45 (3.18, 3.74)	<0.001	3.32 (3.10, 3.56)	<0.001	3.39 (3.13, 3.67)	<0.001
**Gender**												
Male	1.00 (reference)		1.00 (reference)		1.00 (reference)		1.00 (reference)		1.00 (reference)		1.00 (reference)	
Female	1.27 (1.18, 1.36)	<0.001	1.13 (1.03, 1.24)	0.012	1.14 (1.09, 1.20)	<0.001	1.09 (1.03, 1.16)	0.005	1.16 (1.10, 1.21)	<0.001	1.11 (1.04, 1.17)	<0.001
**Race/Ethnicity**												
Non-Black	1.00 (reference)		1.00 (reference)		1.00 (reference)		1.00 (reference)		1.00 (reference)		1.00 (reference)	
Black	1.19 (1.11, 1.27)	<0.001	1.16 (1.05, 1.26)	0.002	1.19 (1.13, 1.25)	<0.001	1.02 (0.96, 1.08)	0.537	1.17 (1.12, 1.23)	<0.001	1.04 (0.98, 1.10)	0.25
**Hispanic/Latino Ethnicity**												
Non-Hispanic	1.00 (reference)		1.00 (reference)		1.00 (reference)		1.00 (reference)		1.00 (reference)		1.00 (reference)	
Hispanic	0.92 (0.85, 1.01)	0.069	0.97 (0.88, 1.08)	0.632	0.50 (0.47, 0.53)	<0.001	0.50 (0.46, 0.54)	<0.001	0.62 (0.58, 0.65)	<0.001	0.62 (0.58, 0.67)	<0.001
**Education level**												
>high school	1.00 (reference)		1.00 (reference)		1.00 (reference)		1.00 (reference)		1.00 (reference)		1.00 (reference)	
≤ High school	1.24 (1.14, 1.34)	<0.001	1.11 (1.01, 1.22)	0.038	1.13 (1.07, 1.19)	<0.001	1.13 (1.07, 1.21)	<0.001	1.15 (1.09, 1.22)	<0.001	1.12 (1.05, 1.19)	<0.001
**Treatment with antihypertensive drugs prior to trial baseline**												
Untreated	1.00 (reference)		1.00 (reference)		1.00 (reference)		1.00 (reference)		1.00 (reference)		1.00 (reference)	
Treated	1.01 (0.90, 1.14)	0.828	0.94 (0.82, 1.08)	0.381	1.08 (1.00, 1.17)	0.055	1.02 (0.93, 1.12)	0.681	1.09 (1.00, 1.17)	0.041	1.02 (0.93, 1.11)	0.709
**Aspirin use (as of 1/1/1999)**												
No	1.00 (reference)		1.00 (reference)		1.00 (reference)		1.00 (reference)		1.00 (reference)		1.00 (reference)	
Yes	0.94 (0.87, 1.01)	0.08	0.94 (0.86, 1.03)	0.178	1.03 (0.98, 1.08)	0.182	0.95 (0.90, 1.01)	0.109	1.01 (0.97, 1.06)	0.571	0.95 (0.90, 1.01)	0.094
**Women taking estrogen at trial baseline** ^†^												
No	1.00 (reference)		-------	--------	1.00 (reference)		-------	--------	1.00 (reference)		-------	--------
Yes	0.84 (0.74, 0.95)	0.005	-------	--------	0.93 (0.86, 1.01)	0.077	-------	--------	0.90 (0.83, 0.98)	0.013	-------	--------
**HDL cholesterol <35 mg/dl (as of 1/1/1999)**												
No	1.00 (reference)		1.00 (reference)		1.00 (reference)		1.00 (reference)		1.00 (reference)		1.00 (reference)	
Yes	0.90 (0.82, 1.00)	0.04	1.02 (0.90, 1.14)	0.785	0.91 (0.85, 0.97)	0.005	0.94 (0.87, 1.01)	0.11	0.91 (0.85, 0.97)	0.003	0.95 (0.88, 1.03)	0.22
**Cigarette smoking at trial baseline**												
Never smoker	1.00 (reference)		1.00 (reference)		1.00 (reference)		1.00 (reference)		1.00 (reference)		1.00 (reference)	
Current smoker	0.81 (0.73, 0.90)	<0.001	1.03 (0.90, 1.17)	0.664	0.93 (0.87, 1.00)	0.037	1.17 (1.07, 1.27)	<0.001	0.92 (0.86, 0.99)	0.019	1.17 (1.07, 1.27)	<0.001
Former smoker	0.85 (0.79, 0.91)	<0.001	0.97 (0.89, 1.07)	0.552	0.97 (0.92, 1.02)	0.168	1.00 (0.94, 1.06)	0.996	0.95 (0.9, 1.00)	0.042	1.01 (0.95, 1.07)	0.84
**Diabetes classification (as of 1/1/1999)**												
Non-diabetes	1.00 (reference)		1.00 (reference)		1.00 (reference)		1.00 (reference)		1.00 (reference)		1.00 (reference)	
Diabetes	1.15 (1.07, 1.24)	<0.001	1.27 (1.17, 1.39)	<0.001	1.21 (1.15, 1.27)	<0.001	1.39 (1.31, 1.47)	<0.001	1.21 (1.16, 1.27)	<0.001	1.38 (1.31, 1.46)	<0.001
**History of Coronary Heart Disease (CHD) (as of 1/1/1999)**												
No	1.00 (reference)		1.00 (reference)		1.00 (reference)		1.00 (reference)		1.00 (reference)		1.00 (reference)	
Yes	1.00 (0.93, 1.08)	0.976	0.95 (0.85, 1.07)	0.426	1.09 (1.03, 1.15)	0.001	0.95 (0.88, 1.02)	0.165	1.08 (1.03, 1.14)	0.002	0.95 (0.88, 1.03)	0.213
**Atherosclerotic Cardiovascular Disease (ASCVD) at trial baseline**												
No	1.00 (reference)		1.00 (reference)		1.00 (reference)		1.00 (reference)		1.00 (reference)		1.00 (reference)	
Yes	1.12 (1.05, 1.20)	0.001	1.24 (1.09, 1.41)	<0.001	1.19 (1.14, 1.25)	<0.001	1.08 (0.99, 1.18)	0.081	1.17 (1.12, 1.23)	<0.001	1.10 (1.01, 1.19)	0.032
**History of myocardial infarction (MI) or stroke (as of 1/1/1999)**												
No	1.00 (reference)		1.00 (reference)		1.00 (reference)		1.00 (reference)		1.00 (reference)		1.00 (reference)	
Yes	1.16 (1.07, 1.25)	<0.001	1.04 (0.92, 1.16)	0.549	1.34 (1.28, 1.41)	<0.001	1.27 (1.18, 1.37)	<0.001	1.32 (1.25, 1.39)	<0.001	1.25 (1.16, 1.34)	<0.001
**History of coronary artery bypass graft (CABG) (as of 1/1/1999)**												
No	1.00 (reference)		1.00 (reference)		1.00 (reference)		1.00 (reference)		1.00 (reference)		1.00 (reference)	
Yes	0.97 (0.88, 1.07)	0.529	1.02 (0.89, 1.17)	0.821	1.13 (1.06, 1.21)	<0.001	1.09 (1.00, 1.19)	0.054	1.10 (1.03, 1.17)	0.004	1.08 (1.00, 1.18)	0.064
**Other ASCVD at trial baseline**												
No	1.00 (reference)		1.00 (reference)		1.00 (reference)		1.00 (reference)		1.00 (reference)		1.00 (reference)	
Yes	1.03 (0.95, 1.11)	0.524	0.88 (0.78, 0.99)	0.033	1.12 (1.07, 1.18)	<0.001	1.08 (1.00, 1.17)	0.04	1.11 (1.05, 1.16)	<0.001	1.06 (0.98, 1.14)	0.13
**Major ST segment depression (as of 1/1/1999)**												
No	1.00 (reference)		1.00 (reference)		1.00 (reference)		1.00 (reference)		1.00 (reference)		1.00 (reference)	
Yes	0.96 (0.84, 1.09)	0.536	0.93 (0.79, 1.10)	0.404	1.00 (0.92, 1.10)	0.912	1.05 (0.95, 1.17)	0.32	1.00 (0.91, 1.08)	0.926	1.04 (0.93, 1.15)	0.514
**Left ventricular hypertrophy (LVH) by Minnesota code (as of 1/1/1999)**												
No/Soft LVH	1.00 (reference)		1.00 (reference)		1.00 (reference)		1.00 (reference)		1.00 (reference)		1.00 (reference)	
Hard LVH	1.18 (0.98, 1.42)	0.082	1.00 (0.81, 1.24)	0.976	1.32 (1.18, 1.49)	<0.001	1.22 (1.07, 1.39)	0.003	1.32 (1.18, 1.49)	<0.001	1.22 (1.07, 1.39)	0.003
**Lipid Lowering Trial (LLT) Participant**												
No	1.00 (reference)		1.00 (reference)		1.00 (reference)		1.00 (reference)		1.00 (reference)		1.00 (reference)	
Yes	0.92 (0.84, 0.99)	0.033	0.93 (0.85, 1.02)	0.106	0.91 (0.86, 0.96)	<0.001	0.96 (0.90, 1.02)	0.165	0.92 (0.87, 0.97)	0.001	0.96 (0.90, 1.02)	0.163
**Obesity (BMI ≥ 30 kg/m2) at trial baseline**												
No	1.00 (reference)		1.00 (reference)		1.00 (reference)		1.00 (reference)		1.00 (reference)		1.00 (reference)	
Yes	0.82 (0.76, 0.88)	<0.001	0.87 (0.80, 0.94)	0.001	0.96 (0.91, 1.00)	0.059	0.99 (0.93, 1.04)	0.609	0.93 (0.89, 0.98)	0.003	0.97 (0.92, 1.02)	0.228
**Blood pressure change (latest BP reading prior to 1/1/1999 minus BP at trial baseline), per 10 mmHg**												
Systolic BP	1.00 (0.98, 1.02)	0.72	0.99 (0.97, 1.02)	0.558	1.01 (1.00, 1.02)	0.132	1.00 (0.98, 1.02)	0.864	1.01 (1.00, 1.03)	0.055	1.00 (0.98, 1.02)	0.942
Diastolic BP	1.03 (1.00, 1.07)	0.077	1.02 (0.97, 1.06)	0.506	1.03 (1.00, 1.05)	0.027	1.02 (0.99, 1.05)	0.285	1.04 (1.01, 1.06)	0.001	1.03 (1.00, 1.06)	0.094

**Abbreviations:** ADRD: Alzheimer’s Disease and Related Dementias; HR: Hazard Ratio; CI: Confidence Interval

Note:

* Adjusted for each covariate shown, in addition to all others presented in the table,

‡ Contrast estimates were garnered from the same model, using a different reference group for randomized group (Chlorthalidone or Amlodipine),

† Estrogen use was evaluated in women only, which prevented simultaneous inclusion of sex and estrogen as covariates

**Table 4:** Hazard ratio (HR) of ADRD by randomized group (3 study drugs) and other factors, calculated by competing risks regression model (1999 to 2017).

Demographic	Alzheimer’s Disease (AD)				Other non-AD Dementias			All ADRD combined				
	Unadjusted HR (95% CI)	P value	Adjusted* HR (95% CI)	P value	Unadjusted HR (95% CI)	P value	Adjusted* HR (95% CI)	P value	Unadjusted HR (95% CI)	P value	Adjusted* HR (95% CI)	P value
**Randomized group**												
Chlorthalidone vs Amlodipine	1.02 (0.94, 1.11)	0.57	1.02 (0.93, 1.12)	0.71	1.02 (0.97, 1.08)	0.47	0.98 (0.92, 1.04)	0.54	1.01 (0.96, 1.07)	0.63	0.98 (0.92, 1.05)	0.57
Lisinopril vs. Amlodipine	1.02 (0.93, 1.12)	0.68	1.00 (0.89, 1.11)	0.95	1.04 (0.97, 1.11)	0.25	0.98 (0.91, 1.05)	0.52	1.03 (0.97, 1.10)	0.28	0.97 (0.90, 1.04)	0.4
Lisinopril vs.	1.00 (0.92, 1.08)	0.92	0.98 (0.89, 1.08)	0.66	1.02 (0.96, 1.08)	0.58	1.00 (0.93, 1.06)	0.91	1.02 (0.97, 1.08)	0.47	0.99 (0.93, 1.05)	0.71
ChlorthalidonE^‡^												
**Age group (as of 1/1/1999)**												
Age <70	1.00 (reference)		1.00 (reference)		1.00 (reference)		1.00 (reference)		1.00 (reference)		1.00 (reference)	
Age 70–79	1.57 (1.44, 1.70)	<0.001	1.61 (1.46, 1.77)	<0.001	1.36 (1.29, 1.44)	<0.001	1.34 (1.27, 1.43)	<0.001	1.37 (1.30, 1.45)	<0.001	1.36 (1.28, 1.44)	<0.001
Age 80+	1.96 (1.77, 2.17)	<0.001	1.92 (1.70, 2.17)	<0.001	1.88 (1.75, 2.02)	<0.001	1.83 (1.68, 1.99)	<0.001	1.93 (1.80, 2.07)	<0.001	1.86 (1.71, 2.01)	<0.001
**Gender**												
Male	1.00 (reference)		1.00 (reference)		1.00 (reference)		1.00 (reference)		1.00 (reference)		1.00 (reference)	
Female	1.41 (1.31, 1.51)	<0.001	1.25 (1.14, 1.36)	<0.001	1.26 (1.20, 1.32)	<0.001	1.19 (1.12, 1.26)	<0.001	1.27 (1.21, 1.33)	<0.001	1.20 (1.13, 1.28)	<0.001
**Race/Ethnicity**												
Non-Black	1.00 (reference)		1.00 (reference)		1.00 (reference)		1.00 (reference)		1.00 (reference)		1.00 (reference)	
Black	1.16 (1.08, 1.25)	<0.001	1.19 (1.09, 1.30)	<0.001	1.17 (1.12, 1.23)	<0.001	1.04 (0.98, 1.11)	0.17	1.15 (1.10, 1.21)	<0.001	1.06 (1.00, 1.12)	0.061
**Hispanic/Latino Ethnicity**												
Non-Hispanic	1.00 (reference)		1.00 (reference)		1.00 (reference)		1.00 (reference)		1.00 (reference)		1.00 (reference)	
Hispanic	1.03 (0.95, 1.12)	0.47	1.08 (0.97, 1.20)	0.16	0.54 (0.50, 0.58)	<0.001	0.54 (0.50, 0.59)	<0.001	0.66 (0.62, 0.71)	<0.001	0.68 (0.63, 0.73)	<0.001
**Education level**												
>high school	1.00 (reference)		1.00 (reference)		1.00 (reference)		1.00 (reference)		1.00 (reference)		1.00 (reference)	
≤ High school	1.12 (1.03, 1.22)	0.006	1.01 (0.92, 1.11)	0.83	1.04 (0.98, 1.10)	0.17	1.04 (0.98, 1.11)	0.2	1.06 (1.01, 1.12)	0.022	1.03 (0.97, 1.10)	0.32
**Treatment with antihypertensive drugs prior to trial baseline**												
Untreated	1.00 (reference)		1.00 (reference)		1.00 (reference)		1.00 (reference)		1.00 (reference)		1.00 (reference)	
Treated	0.96 (0.86, 1.08)	0.51	0.91 (0.80, 1.04)	0.17	1.03 (0.95, 1.12)	0.43	0.98 (0.89, 1.07)	0.64	1.03 (0.95, 1.12)	0.42	0.97 (0.89, 1.07)	0.57
**Aspirin use (as of 1/1/1999)**												
No	1.00 (reference)		1.00 (reference)		1.00 (reference)		1.00 (reference)		1.00 (reference)		1.00 (reference)	
Yes	0.90 (0.84, 0.97)	0.004	0.95 (0.87, 1.04)	0.3	1.00 (0.95, 1.05)	0.93	0.97 (0.92, 1.03)	0.35	0.98 (0.93, 1.03)	0.37	0.97 (0.91, 1.03)	0.29
**Women taking estrogen at trial baseline** ^†^												
No	1.00 (reference)		-------	--------	1.00 (reference)		-------	--------	1.00 (reference)		-------	--------
Yes	0.97 (0.86, 1.09)	0.58	-------	--------	1.06 (0.97, 1.14)	0.18	-------	--------	1.03 (0.95, 1.11)	0.53	-------	--------
**HDL cholesterol <35 mg/dl (as of 1/1/1999)**												
No	1.00 (reference)		1.00 (reference)		1.00 (reference)		1.00 (reference)		1.00 (reference)		1.00 (reference)	
Yes	0.84 (0.76, 0.92)	<0.001	0.96 (0.86, 1.09)	0.55	0.85 (0.79, 0.91)	<0.001	0.90 (0.83, 0.97)	0.006	0.85 (0.80, 0.91)	<0.001	0.91 (0.85, 0.99)	0.02
**Cigarette smoking at trial baseline**												
Never smoker	1.00 (reference)		1.00 (reference)		1.00 (reference)		1.00 (reference)		1.00 (reference)		1.00 (reference)	
Current smoker	0.66 (0.59, 0.74)	<0.001	0.74 (0.65, 0.84)	<0.001	0.77 (0.72, 0.83)	<0.001	0.84 (0.78, 0.92)	<0.001	0.77 (0.72, 0.82)	<0.001	0.85 (0.79, 0.93)	<0.001
Former smoker	0.77 (0.72, 0.83)	<0.001	0.90 (0.82, 0.99)	0.024	0.88 (0.84, 0.92)	<0.001	0.92 (0.87, 0.98)	0.008	0.87 (0.83, 0.91)	<0.001	0.93 (0.88, 0.99)	0.021
**Diabetes classification (as of 1/1/1999)**												
Non-diabetes	1.00 (reference)		1.00 (reference)		1.00 (reference)		1.00 (reference)		1.00 (reference)		1.00 (reference)	
Diabetes	0.99 (0.93, 1.07)	0.88	1.02 (0.93, 1.11)	0.7	1.07 (1.02, 1.12)	0.009	1.13 (1.07, 1.20)	<0.001	1.07 (1.02, 1.13)	0.004	1.14 (1.07, 1.20)	<0.001
**History of Coronary Heart Disease (CHD) (as of 1/1/1999)**												
No	1.00 (reference)		1.00 (reference)		1.00 (reference)		1.00 (reference)		1.00 (reference)		1.00 (reference)	
Yes	0.90 (0.83, 0.97)	0.006	0.97 (0.86, 1.09)	0.63	0.99 (0.94, 1.04)	0.73	0.97 (0.89, 1.05)	0.39	0.99 (0.94, 1.04)	0.58	0.97 (0.90, 1.05)	0.46
**Atherosclerotic Cardiovascular Disease (ASCVD) at trial baseline**												
No	1.00 (reference)		1.00 (reference)		1.00 (reference)		1.00 (reference)		1.00 (reference)		1.00 (reference)	
Yes	1.04 (0.97, 1.11)	0.3	1.27 (1.11, 1.44)	<0.001	1.11 (1.06, 1.16)	<0.001	1.07 (0.98, 1.17)	0.12	1.10 (1.05, 1.15)	<0.001	1.09 (1.00, 1.19)	0.045
**History of Myocardial Infarction (MI) or stroke (as of 1/1/1999)**												
No	1.00 (reference)		1.00 (reference)		1.00 (reference)		1.00 (reference)		1.00 (reference)		1.00 (reference)	
Yes	0.93 (0.86, 1.01)	0.095	0.86 (0.76, 0.96)	0.008	1.12 (1.06, 1.18)	<0.001	1.08 (1.00, 1.17)	0.043	1.10 (1.05, 1.16)	<0.001	1.07 (0.99, 1.15)	0.11
**History of Coronary Artery Bypass Graft (CABG) (as of 1/1/1999)**												
No	1.00 (reference)		1.00 (reference)		1.00 (reference)		1.00 (reference)		1.00 (reference)		1.00 (reference)	
Yes	0.85 (0.77, 0.94)	0.001	0.92 (0.80, 1.06)	0.24	1.00 (0.94, 1.07)	0.94	1.00 (0.91, 1.09)	0.93	0.97 (0.91, 1.04)	0.42	0.99 (0.91, 1.08)	0.86
**Other ASCVD at trial baseline**												
No	1.00 (reference)		1.00 (reference)		1.00 (reference)		1.00 (reference)		1.00 (reference)		1.00 (reference)	
Yes	0.98 (0.90, 1.06)	0.55	0.80 (0.71, 0.90)	<0.001	1.08 (1.02, 1.14)	0.004	1.01 (0.93, 1.09)	0.87	1.06 (1.01, 1.12)	0.019	0.98 (0.91, 1.06)	0.69
**Major ST segment depression (as of 1/1/1999)**												
No	1.00 (reference)		1.00 (reference)		1.00 (reference)		1.00 (reference)		1.00 (reference)		1.00 (reference)	
Yes	0.91 (0.80, 1.04)	0.17	0.83 (0.70, 0.97)	0.02	0.97 (0.89, 1.06)	0.46	0.94 (0.84, 1.05)	0.26	0.96 (0.88, 1.05)	0.35	0.93 (0.83, 1.03)	0.15
**Left ventricular hypertrophy (LVH) by Minnesota code (as of 1/1/1999)**												
No/Soft LVH	1.00 (reference)		1.00 (reference)		1.00 (reference)		1.00 (reference)		1.00 (reference)		1.00 (reference)	
Hard LVH	0.93 (0.77, 1.13)	0.46	0.81 (0.65, 1.01)	0.065	1.09 (0.96, 1.23)	0.19	1.05 (0.91, 1.21)	0.54	1.10 (0.97, 1.24)	0.13	1.06 (0.92, 1.22)	0.43
**Lipid Lowering Trial (LLT) Participant**												
No	1.00 (reference)		1.00 (reference)		1.00 (reference)		1.00 (reference)		1.00 (reference)		1.00 (reference)	
Yes	0.97 (0.90, 1.05)	0.47	0.96 (0.88, 1.05)	0.39	0.95 (0.90, 1.01)	0.084	0.98 (0.93, 1.05)	0.61	0.96 (0.91, 1.01)	0.14	0.98 (0.93, 1.04)	0.58
**Obesity (BMI ≥ 30 kg/m2) at trial baseline**												
No	1.00 (reference)		1.00 (reference)		1.00 (reference)		1.00 (reference)		1.00 (reference)		1.00 (reference)	
Yes	0.85 (0.80, 0.92)	<0.001	0.86 (0.79, 0.93)	<0.001	0.99 (0.94, 1.03)	0.56	0.97 (0.92, 1.03)	0.29	0.96 (0.91, 1.00)	0.068	0.95 (0.90, 1.00)	0.068
**Blood pressure change (latest BP reading prior to 1/1/1999 minus BP at trial baseline), per 10 mmHg**												
Systolic BP	1.00 (0.98, 1.02)	0.86	0.99 (0.96, 1.02)	0.41	1.01 (0.99, 1.02)	0.29	1.00 (0.98, 1.02)	0.81	1.01 (1.00, 1.02)	0.14	1.00 (0.98, 1.02)	0.83
Diastolic BP	1.03 (0.99, 1.06)	0.13	1.02 (0.98, 1.07)	0.34	1.02 (1.00, 1.04)	0.082	1.02 (0.99, 1.05)	0.23	1.03 (1.01, 1.05)	0.007	1.03 (1.00, 1.06)	0.075

**Abbreviations:** ADRD: Alzheimer’s Disease and Related Dementias; HR: Hazard Ratio; CI: Confidence Interval

Note:

* Adjusted for each covariate shown, in addition to all others presented in the table,

‡ Contrast estimates were garnered from the same model, using a different reference group for randomized group (Chlorthalidone or Amlodipine),

† Estrogen use was evaluated in women only, which prevented simultaneous inclusion of sex and estrogen as covariates.

## Data Availability

The ALLHAT data and Medicare claims data are not public-use datasets. However, researchers may request the ALLHAT data with the approval from the ALLHAT Coordinating Center in Houston and the Medicare claims data with the approval from the Center for Medicare and Medicaid Services (CMS). We plan to share the statistical models and statistical programs that we used to analyze these data upon request.
