# Supplementary material for: Risk of Developing Alzheimer’s Disease and Related Dementias in ALLHAT Trial Participants Receiving Diuretic, ACE-Inhibitor, or Calcium-Channel Blocker with 18 Years of Follow-Up
Source: J Alzheimers Dis Parkinsonism. Author manuscript; Available in PMC 2022 May 12. (PMC9095428)
Supplement: Supplement File [file NIHMS1803876-supplement-Supplement_File.pdf]

Supplemental Materials:

Table S1. Antihypertensive Medication Classes and Common Generic and Brand Names

#	**Antihypertensive medication classes**<br>**FDA Chronic Antihypertensive Treatment Classes**	**Generic Name (GNN)**	**Brand Name (BN)***
1	Aldosterone antagonists[†]	Eplerenone<br>Spironolactone	Inspra<br>Aldactone, Carospir, Aldactazide[‡]
2	Alpha 1 adrenergic receptor agonist (selective; **α-blocker**)	**Doxazosin [Step 1 in ALLHAT]**<br>Prazosin<br>Terazosin	Cardura (XL)<br>Minipress, Prazin, Prazo<br>Hytrin
3	Angiotensin-converting enzyme (**ACE**) inhibitor	Benazepril<br>Captopril<br>Enalapril<br>Fosinopril<br>**Lisinopril [Step I]**<br>Moexipril<br>Perindopril<br>Quinapril<br>Ramipril<br>Trandolapril	Lotensin, Lotensin HCT[‡], Lotrel[‡]<br>Capoten, Captoril, Capozide[‡]<br>Vasotec, Enalaprilat, Epaned, Lexxel[‡§], Vaseretic[‡]<br>Monopril<br>Prinivil, Qbrelis, Zestril, Zestoretic[‡]<br>Univasc, Uniretic[‡]<br>Aceon, Prestalia[‡]<br>Accupril, Accuretic[‡]<br>Altace<br>Mavik, Tarka[‡]
4	Angiotensin II receptor blockers (**ARB**)	Azilsartan<br>Candesartan<br>Eprosartan<br>Irbesartan<br>Losartan<br>Olmesartan<br>Telmisartan<br>Valsartan	Edarbi, Edarbychlor[‡]<br>Atacand, Atacand HCT[‡]<br>Teveten, Teveten HCT[‡], Teveten Plus[‡]<br>Avapro, Avalide[‡]<br>Cozaar, Hyzaar[‡]<br>Benicar, Azor[‡], Benicar HCT[‡], Tribenzor[‡]<br>Micardis, Micardis HCT[‡], Micardis Plus[‡],Twynsta[‡]<br>Diovan, Prexxartan[§], Byvalson[‡], Diovan HCT[‡], Exforge HCT[‡], Entresto[‡], Valturna[‡]
5	Arteriolar vasodilators	**Hydralazine [Step 3 in ALLHAT]**<br>Minoxidil	-, Hydra-zide[‡]<br>Loniten, Minodyl, Minoxidil HTN
6	Autonomic ganglionic vasodilators	Mecamylamine	-
7	Beta adrenergic blockers (**β-blockers**)	Acebutolol<br>**Atenolol [Step 2 in ALLHAT]**<br>Betaxolol<br>Bisoprolol<br>Carvedilol {*also with α1 properties*}<br>Esmolol[Π]<br>Labetalol {*also with α1 properties*}<br>Metoprolol<br><br>Nadolol<br>Nebivolol<br>Penbutolol<br>Propranolol<br>Sotalol[Π]	Sectral<br>Tenormin, Tenoretic[‡]<br>Kerlone<br>Monocor, Zebeta, Ziac[‡]<br>Coreg, Coreg CR<br>Brevibloc[Π]<br>Trandate<br>Kapspargo Sprinkle, Lopressor, Toprol XL, Dutoprol[‡], Lopressor HCT[‡]<br>Corgard, Corzide[‡]<br>Bystolic, Byvalson[‡]<br>Levatol<br>Inderal, Inderal LA, InnoPran XL, Inderide[‡]<br>Betapace, Sorine[Π]
8	Calcium channel blocker (CCB)- dihydropyridine	**Amlodipine [Step I]**<br><br>Felodipine	Norvasc, Amturnide[‡], Azor[‡], Exforge HCT[‡], Lotrel[‡], Prestalia[‡], Tekamlo[‡], Tribenzor[‡], Twynsta[‡]

		 <br>Isradipine<br>Nicardipine<br>Nifedipine<br><br>Nimodipine<br>Nisoldipine	Cabren, Cardioplen XL, Felendil XL, Felogen XL, Felotens XL, Keloc SR, Neofel XL, Plendil, Renedil, Vascalpha, Lexxel‡§<br>-<br>Cardene, Cardene SR<br>Adalat, Adalat CC, Afeditab CR, Nifediac CC, Nifedical XL, Procardia, Procardia XL<br><br>Sular
9	Calcium channel blocker – non-dihydropyridine	Diltiazem<br><br><br>Verapamil	Cardizem, Cardizem CD, Cardizem LA, Dilacor, Dilacor XR, Dilatrate, Diltazem, Diltazem CD, Diltiaz, Diltiaz CD, Diltiaz SR, Tiazac<br>Calan, Calan HS, Calan SR, Covera HS, Isoptin, Isoptin SR, Verelan, Verelan PM, Tarka‡
10	Central alpha 2 adrenergic agonists	**Clonidine [Step 2]**<br>Guanabenz§<br>Guanfacine<br>Methyldopa	Catapres, Jenloga, Kapvay, Nexiclon XR, Clorpres‡<br>Wytensin§<br>Intuniv, Tenex<br>Aldomet
11	Diuretics: thiazide	Bendroflumenthiazide<br>Chlorothiazide<br>Hydrochlorothiazide	Naturetin§, NeoNaClex§, Urizide§, Corzide‡<br>Diuril, Tekturna HCT‡<br>Microzide, Accuretic‡, Aldactazide‡, Atacand HCT‡, Avalide‡, Benicar HCT‡, Capozide‡, Corzide‡, Dutoprol‡, Exforge HCT‡, Hydra-zide‡, Hyzaar‡, Inderide‡, Lopressor HCT‡, Lotensin HCT‡, Maxide‡, Micardis HCT‡, Micardis Plus‡, Moduretic‡, Teveten HCT‡, Teveten Plus‡, Tribenzor‡, Uniretic‡, Vaseretic‡, Zestoretic‡, Ziac‡
12	Diuretics: thiazide-type	**Chlorthalidone [Step I]**<br><br>Indapamide<br>Metolazone	Hygroton, Thalitone, Chlorthalid, Clorpres‡, Edarbychlor‡, Tenoretic‡<br>-<br>Zaroxolyn
13	Diuretics: loop	Bumetanide<br>Ethacrynic acidΠ<br>Furosemide<br>Torsemide	Bumex, Burinex<br>EdecrinΠ<br>Lasix<br>Demadex
14	Diuretics: potassium-sparing†	Amiloride<br>Triamterene	Midamor, Moduretic‡<br>Dyrenium, Maxide‡
15	Peripheral adrenergic neuron antagonist†	**Reserpine [Step 2]**	Serpasil
16	Renin inhibitors	Aliskiren	Tekturna, Tekturna HCT‡, Teklamo‡, Amturnide‡, Valturna‡

*Part-D data appear to have generic name (GNN) only because BN has the same info as GNN in the dataset (so far as noted).
†Not used alone/used in combination for chronic hypertension treatment
‡Combination medication also listed in another class
§Currently discontinued in the US but in use during ALLHAT

Table S2. ICD-9 and ICD-10 codes for Alzheimer’s Disease and related Dementias (ADRD)

ICD-9	Description for ICD-9 codes*	ICD-10	Description for ICD-10 codes*
290.0	Senile dementia, uncomplicated	F03.90	Unspecified dementia without behavioral disturbance
290.1	Presenile dementia, uncomplicated	F03.90	Unspecified dementia without behavioral disturbance
290.2	Senile dementia with delusional or depressive features	F03.90	Unspecified dementia without behavioral disturbance
290.3	Senile dementia with delirium (acute confusional state)	F03.90	Unspecified dementia without behavioral disturbance
290.4	Vascular dementia	F01.5	Vascular dementia
290.8	Other specified senile psychotic conditions	F03.90	Unspecified dementia without behavioral disturbance
290.9	Unspecified senile psychotic condition	F03.90	Unspecified dementia without behavioral disturbance
291.2	Alcohol-induced persisting dementia	F10.27	Alcohol dependence with alcohol-induced dementia
292.82	Drug-induced persisting dementia	F19.97	Other psychoactive substance use, unspecified with psychoactive substance-induced persisting dementia
294.10	Dementia in conditions classified elsewhere without behavioral disturbance	F02.80	Dementia in other diseases classified elsewhere without behavioral disturbance
294.11	Dementia in conditions classified elsewhere with behavioral disturbance	F02.81	Dementia in other diseases classified elsewhere with behavioral disturbance
294.20	Dementia, unspecified, without behavioral disturbance	F03.90	Unspecified dementia without behavioral disturbance
294.21	Dementia, unspecified, with behavioral disturbance	F03.91	Unspecified dementia with behavioral disturbance
294.8	Other persistent mental disorders due to conditions classified elsewhere	F06.0	Psychotic disorder with hallucinations due to known physiological condition
294.9	Unspecified persistent mental disorders due to conditions classified elsewhere	F06.8	Other specified mental disorders due to known physiological condition
331.0	Alzheimer's disease	G30	Alzheimer's disease
		G30.0	Alzheimer's disease with early onset
		G30.1	Alzheimer's disease with late onset
		G30.8	Other Alzheimer's disease
		G30.9	Alzheimer's disease, unspecified
331.11	Frontotemporal dementia, Pick's disease	G31.01	Frontotemporal dementia, Pick's disease
331.19	Other frontotemporal dementia	G31.09	Other frontotemporal dementia
331.2	Senile degeneration of brain	G31.1	Senile degeneration of brain, not elsewhere classified
331.7	Cerebral degeneration in dis. classified elsewhere	G94	Other disorders of brain in dis. classified elsewhere
331.82	Dementia with Lewy bodies	G31.83	Dementia with Lewy bodies
331.83	Mild cognitive impairment, so stated	G31.84	Mild cognitive impairment, so stated
331.89	Other cerebral degeneration	G31.89	Other specified degenerative dis. of nervous system
331.9	Cerebral degeneration, unspecified	G31.9	Degenerative disease of nervous system, unspecified
797	Senility without mention of psychosis	R41.81	Age-related cognitive decline

* ICD-9/10-CM: International Classification of Diseases, 9th/10th Revision, Clinical Modification.

Figure S1. Kaplan-Meier cumulative incidence of Alzheimer's Disease (AD) (S1a), non-AD Dementias (S1b), and any ADRD (S1c) identified from any diagnosis codes (that occurred at least twice with at least 30 days apart) from 1999 to 2017

S1a. Alzheimer's Disease (AD)

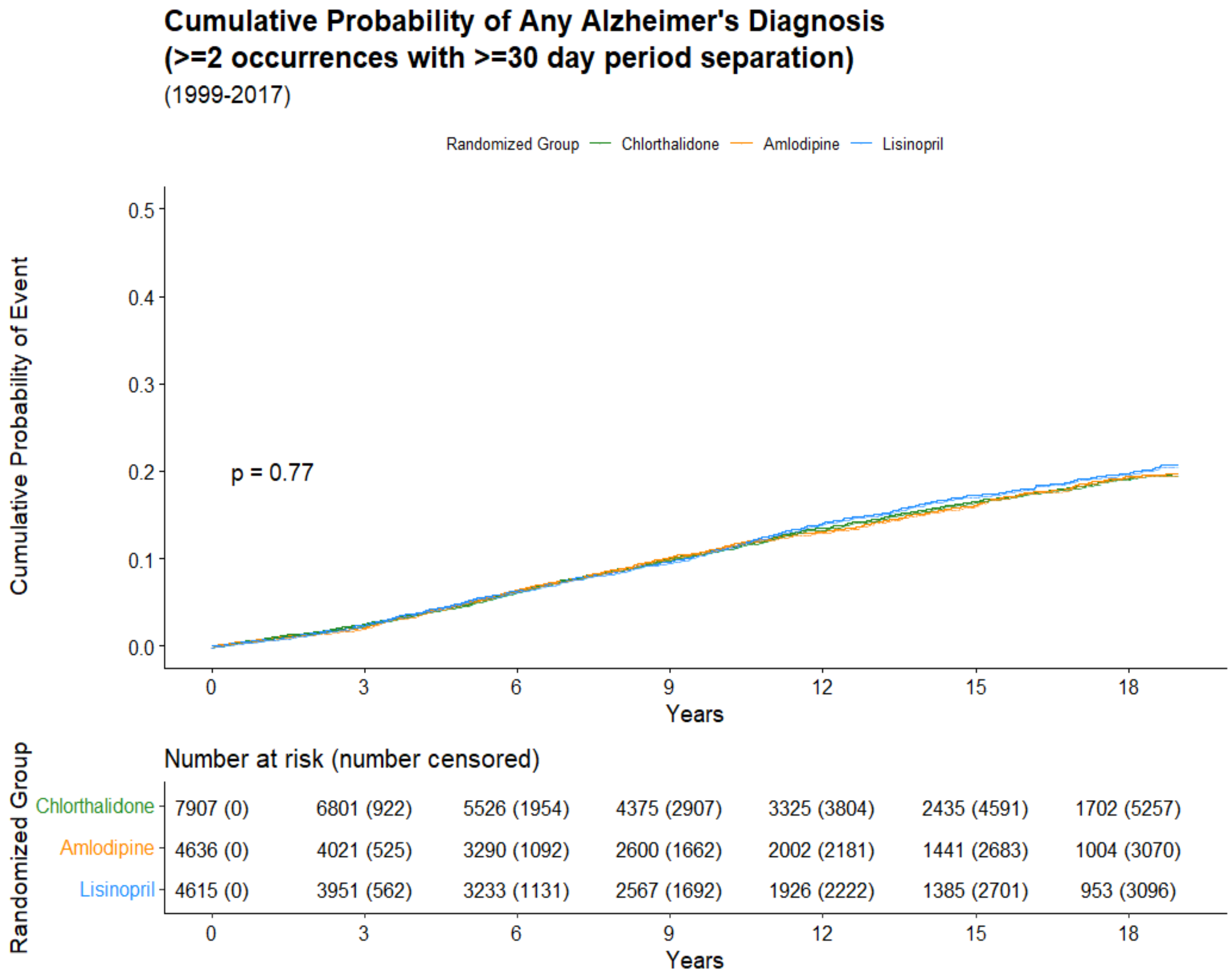

S1b. non-AD Dementias

Cumulative Probability of Any Non-Alzheimer's Dementia Diagnosis (>=2 occurrences with >=30 day period separation)

(1999-2017)

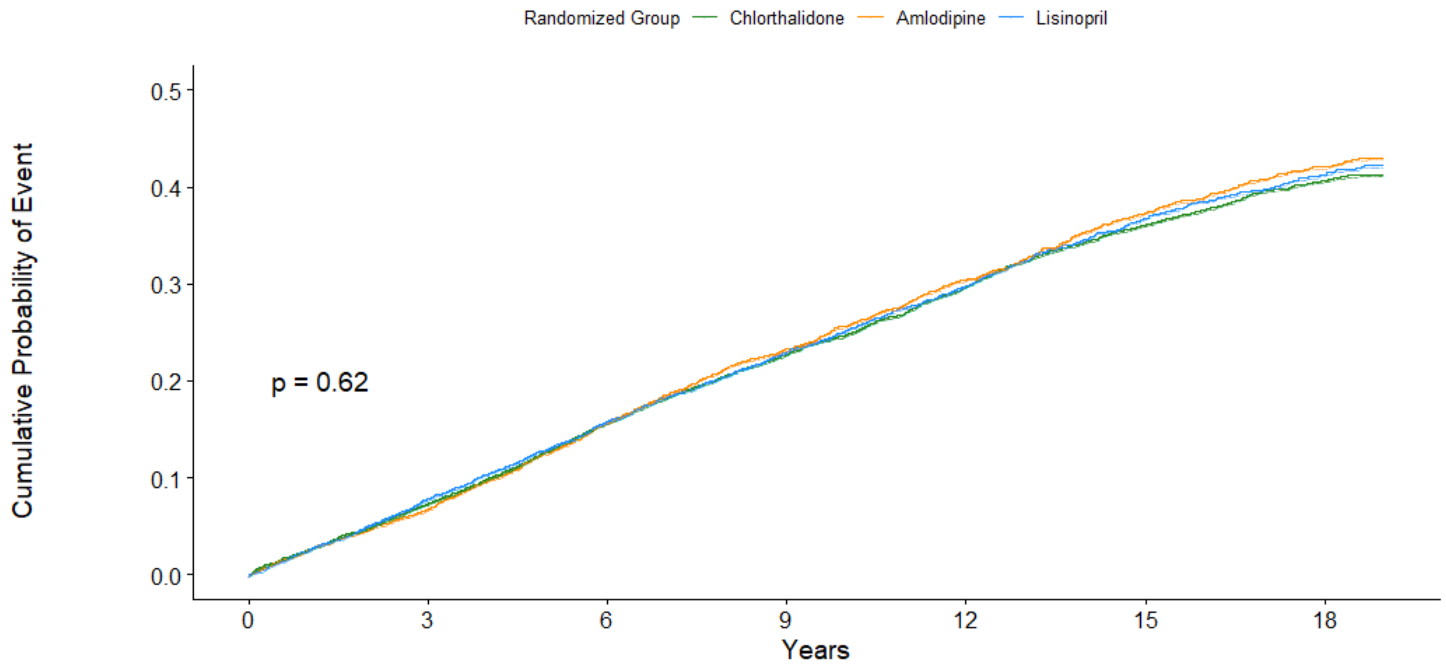

Number at risk (number censored)

Randomized Group	0	3	6	9	12	15	18
Chlorthalidone	7907 (0)	6498 (859)	5077 (1739)	3907 (2518)	2871 (3235)	2037 (3836)	1423 (4321)
Amlodipine	4636 (0)	3849 (491)	3031 (965)	2332 (1411)	1724 (1821)	1198 (2196)	826 (2487)
Lisinopril	4615 (0)	3751 (524)	2961 (1013)	2282 (1458)	1697 (1861)	1184 (2222)	827 (2506)

Years

S1c. Any ADRD (Alzheimer's Disease or Related Dementias)

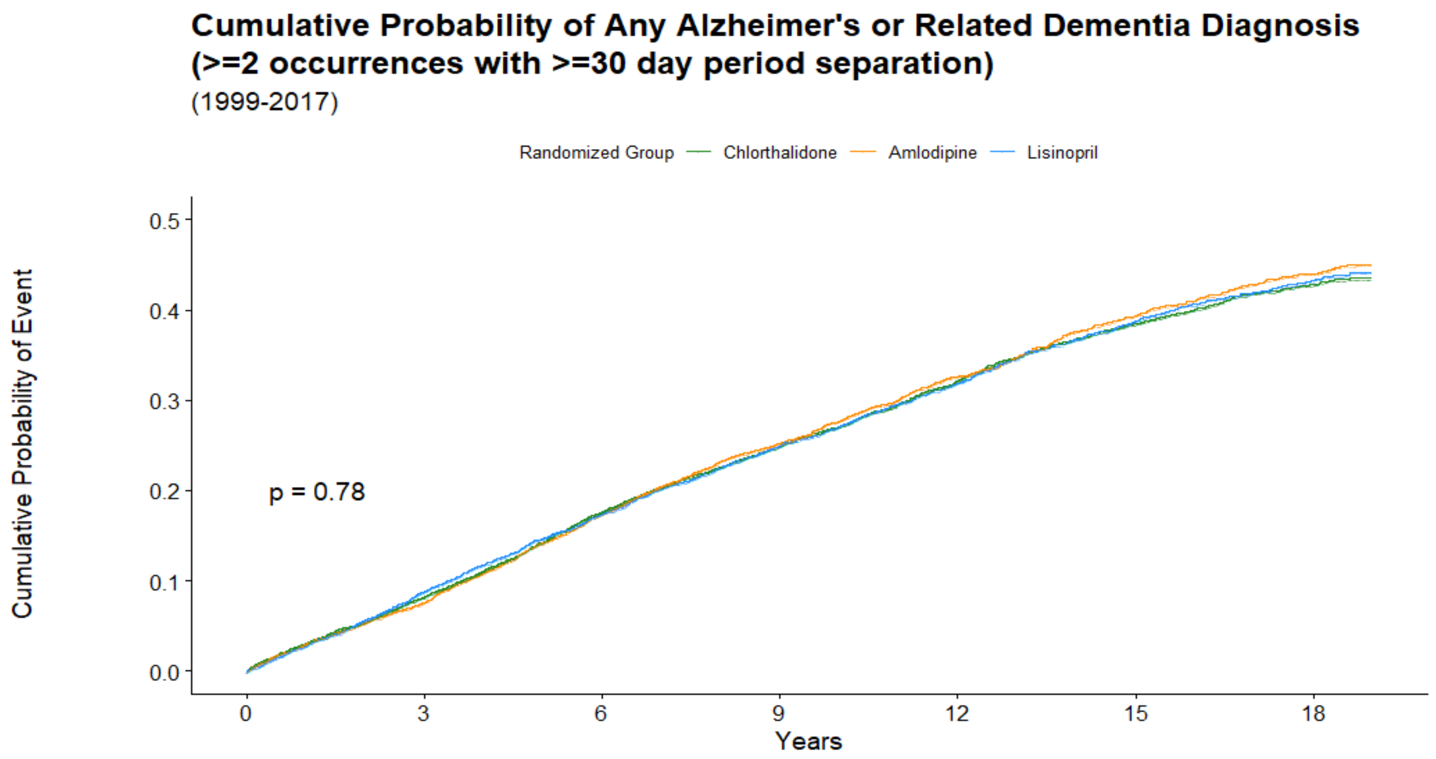

Number at risk (number censored)

Randomized Group	0	3	6	9	12	15	18
Chlorthalidone	7907 (0)	6449 (842)	4982 (1693)	3831 (2442)	2803 (3134)	1992 (3711)	1394 (4184)
Amlodipine	4636 (0)	3816 (487)	2982 (943)	2287 (1373)	1682 (1773)	1171 (2135)	812 (2414)
Lisinopril	4615 (0)	3717 (517)	2914 (991)	2233 (1425)	1654 (1818)	1152 (2170)	807 (2441)

Years

Figure S2. Kaplan-Meier cumulative incidence of Alzheimer's Disease (AD) (S2a), non-AD Dementias (S2b), and any ADRD (S2c) identified from primary diagnosis codes only (that occurred at least twice with at least 30 days apart) from 1999 to 2017

S2a. Alzheimer's Disease (AD)

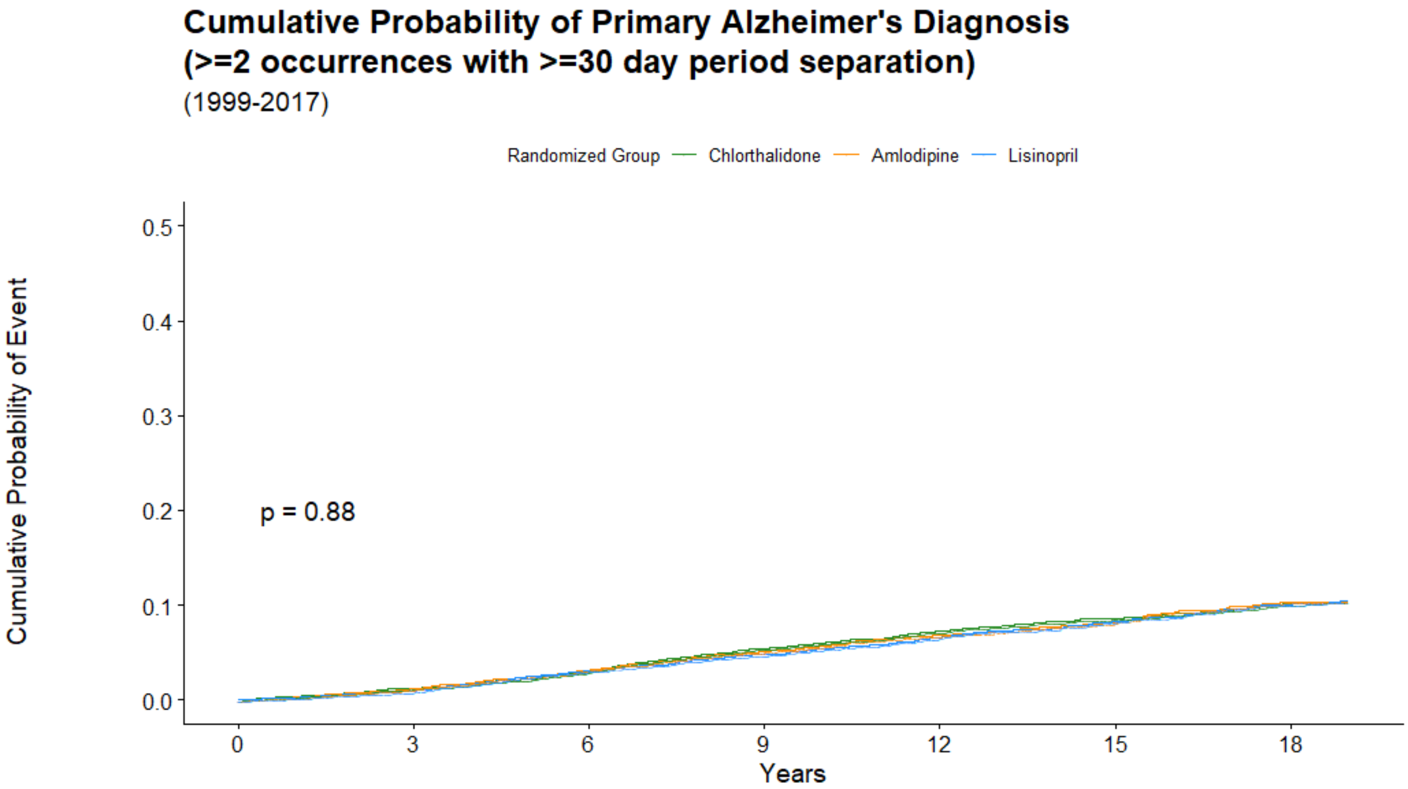

Number at risk (number censored)

Randomized Group	0	3	6	9	12	15	18
Chlorthalidone	7907 (0)	6876 (942)	5672 (2033)	4517 (3058)	3453 (4045)	2528 (4925)	1773 (5644)
Amlodipine	4636 (0)	4053 (534)	3369 (1141)	2689 (1759)	2079 (2326)	1514 (2865)	1037 (3310)
Lisinopril	4615 (0)	4005 (572)	3320 (1174)	2659 (1782)	2030 (2368)	1465 (2900)	1001 (3340)

Years

S2b. non-AD Dementias

Cumulative Probability of Primary Non-Alzheimer's Dementia Diagnosis (>=2 occurrences with >=30 day period separation)

(1999-2017)

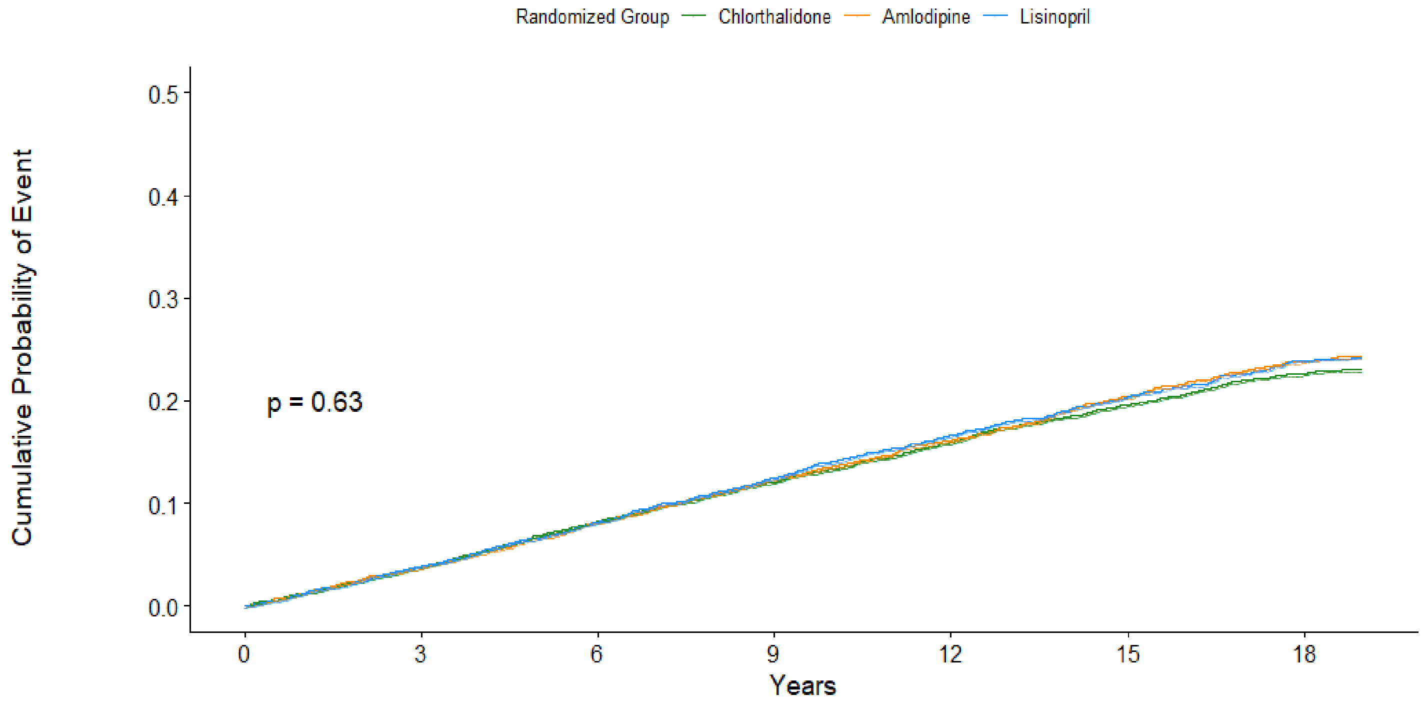

Number at risk (number censored)

Randomized Group	0	3	6	9	12	15	18
Chlorthalidone	7907 (0)	6721 (908)	5418 (1920)	4265 (2867)	3226 (3740)	2334 (4508)	1634 (5130)
Amlodipine	4636 (0)	3950 (523)	3226 (1077)	2540 (1628)	1941 (2131)	1384 (2603)	954 (2981)
Lisinopril	4615 (0)	3895 (552)	3174 (1112)	2499 (1650)	1893 (2150)	1354 (2615)	923 (2996)

Years

S2c. Any ADRD (Alzheimer’s Disease or Related Dementias)

Cumulative Probability of Primary Alzheimer's or Related Dementia Diagnosis (>=2 occurrences with >=30 day period separation)

(1999-2017)

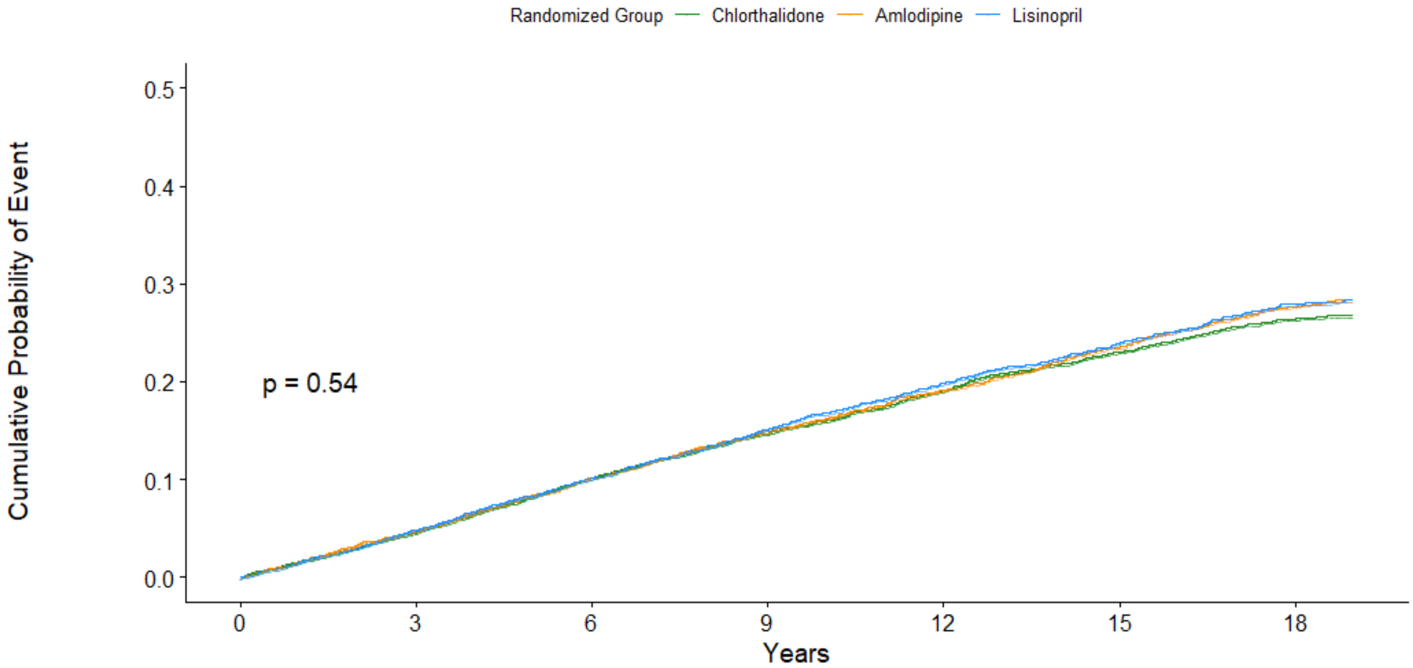

Number at risk (number censored)

Randomized Group	0	3	6	9	12	15	18
Chlorthalidone	7907 (0)	6666 (900)	5332 (1874)	4181 (2778)	3155 (3612)	2286 (4345)	1599 (4943)
Amlodipine	4636 (0)	3913 (516)	3169 (1052)	2491 (1577)	1892 (2066)	1347 (2521)	929 (2874)
Lisinopril	4615 (0)	3859 (549)	3118 (1088)	2437 (1610)	1837 (2089)	1313 (2531)	899 (2886)

Years

Table S3: Hazard ratio (HR) of Any Diagnosis of ADRD (with ≥2 occurrences with 30-day period of separation) by Randomized Group (3 Study Drugs) and Other Factors (1999 to 2017), Calculated by Standard Cox Proportional Hazards Model or by Competing Risks Regression (CRR) Model

Demographic	Alzheimer's Disease (AD)				Other non-AD Dementias				All ADRD combined			
	Adjusted Standard HR (95% CI)	P value	Adjusted CRR HR (95% CI)	P value	Adjusted Standard HR (95% CI)	P value	Adjusted CRR HR (95% CI)	P value	Adjusted Standard HR (95% CI)	P value	Adjusted CRR HR (95% CI)	P value
**Randomized group**												
Chlorthalidone vs Amlodipine	1.07 (0.95, 1.21)	0.264	1.05 (0.93, 1.18)	0.420	0.94 (0.87, 1.02)	0.142	0.92 (0.85, 1.00)	0.041	0.97 (0.90, 1.04)	0.402	0.95 (0.88, 1.02)	0.160
Lisinopril vs Amlodipine	1.06 (0.93, 1.21)	0.383	1.04 (0.91, 1.19)	0.560	0.98 (0.90, 1.07)	0.599	0.96 (0.88, 1.05)	0.420	0.99 (0.91, 1.08)	0.896	0.98 (0.90, 1.06)	0.610
Lisinopril vs Chlorthalidone‡	0.99 (0.88, 1.12)	0.892	0.99 (0.88, 1.12)	0.890	1.04 (0.96, 1.12)	0.389	1.05 (0.97, 1.13)	0.270	1.03 (0.95, 1.11)	0.495	1.03 (0.96, 1.11)	0.410
**Age group (as of 1/1/1999)**												
Age <70	1.00 (reference)		1.00 (reference)		1.00 (reference)		1.00 (reference)		1.00 (reference)		1.00 (reference)	
Age 70-79	2.03 (1.80, 2.29)	<0.001	1.65 (1.46, 1.85)	<0.001	1.70 (1.58, 1.84)	<0.001	1.40 (1.30, 1.50)	<0.001	1.72 (1.60, 1.85)	<0.001	1.42 (1.32, 1.53)	<0.001
Age 80+	3.52 (3.01, 4.11)	<0.001	1.75 (1.50, 2.03)	<0.001	3.34 (3.02, 3.69)	<0.001	1.72 (1.55, 1.90)	<0.001	3.36 (3.05, 3.69)	<0.001	1.80 (1.63, 1.98)	<0.001
**Gender**												
Male	1.00 (reference)		1.00 (reference)		1.00 (reference)		1.00 (reference)		1.00 (reference)		1.00 (reference)	
Female	1.23 (1.10, 1.38)	<0.001	1.36 (1.21, 1.52)	<0.001	1.12 (1.04, 1.20)	0.004	1.22 (1.13, 1.31)	<0.001	1.14 (1.06, 1.22)	<0.001	1.24 (1.15, 1.33)	<0.001
**Race/Ethnicity**												
Non-Black	1.00 (reference)		1.00 (reference)		1.00 (reference)		1.00 (reference)		1.00 (reference)		1.00 (reference)	
Black	1.24 (1.11, 1.38)	<0.001	1.26 (1.13, 1.41)	<0.001	1.02 (0.95, 1.10)	0.628	1.04 (0.97, 1.12)	0.300	1.02 (0.95, 1.10)	0.521	1.04 (0.97, 1.12)	0.240
**Hispanic/Latino Ethnicity**												
Non-Hispanic	1.00 (reference)		1.00 (reference)		1.00 (reference)		1.00 (reference)		1.00 (reference)		1.00 (reference)	
Hispanic	0.81 (0.71, 0.93)	0.003	0.90 (0.78, 1.03)	0.130	0.42 (0.38, 0.47)	<0.001	0.46 (0.41, 0.52)	<0.001	0.54 (0.49, 0.60)	<0.001	0.60 (0.54, 0.66)	<0.001
**Education level**												
>High school	1.00 (reference)		1.00 (reference)		1.00 (reference)		1.00 (reference)		1.00 (reference)		1.00 (reference)	
≤High school	1.11 (0.98, 1.24)	0.094	1.01 (0.90, 1.13)	0.890	1.10 (1.02, 1.19)	0.011	1.01 (0.94, 1.09)	0.800	1.09 (1.02, 1.18)	0.017	1.00 (0.93, 1.08)	0.910
**Treatment with antihypertensive drugs prior to trial baseline**												
Untreated	1.00 (reference)		1.00 (reference)		1.00 (reference)		1.00 (reference)		1.00 (reference)		1.00 (reference)	
Treated	0.87 (0.74, 1.02)	0.089	0.84 (0.72, 0.99)	0.037	0.97 (0.87, 1.09)	0.615	0.94 (0.84, 1.05)	0.260	0.97 (0.87, 1.09)	0.626	0.94 (0.84, 1.05)	0.280
**Aspirin use (as of 1/1/1999)**												
No	1.00 (reference)		1.00 (reference)		1.00 (reference)		1.00 (reference)		1.00 (reference)		1.00 (reference)	
Yes	0.98 (0.87, 1.09)	0.671	0.99 (0.89, 1.11)	0.890	0.95 (0.88, 1.02)	0.126	0.96 (0.90, 1.04)	0.320	0.94 (0.88, 1.01)	0.093	0.96 (0.89, 1.03)	0.240
**HDL cholesterol <35 mg/dl (as of 1/1/1999)**												
No	1.00 (reference)		1.00 (reference)		1.00 (reference)		1.00 (reference)		1.00 (reference)		1.00 (reference)	
Yes	1.02 (0.88, 1.18)	0.793	0.96 (0.83, 1.12)	0.610	0.92 (0.84, 1.02)	0.106	0.88 (0.80, 0.97)	0.012	0.93 (0.85, 1.03)	0.155	0.89 (0.82, 0.98)	0.019
**Cigarette smoking at trial baseline**												
Never smoker	1.00 (reference)		1.00 (reference)		1.00 (reference)		1.00 (reference)		1.00 (reference)		1.00 (reference)	
Current smoker	0.90 (0.76, 1.06)	0.209	0.66 (0.56, 0.77)	<0.001	1.10 (0.99, 1.22)	0.088	0.80 (0.72, 0.89)	<0.001	1.06 (0.96, 1.18)	0.233	0.79 (0.71, 0.87)	<0.001

Former smoker	0.99 (0.88, 1.10)	0.812	0.91 (0.82, 1.02)	0.099	0.98 (0.91, 1.06)	0.628	0.92 (0.85, 0.99)	0.021	0.98 (0.91, 1.05)	0.523	0.91 (0.85, 0.98)	0.012
**Diabetes classification (as of 1/1/1999)**												
Non-diabetes	1.00 (reference)		1.00 (reference)		1.00 (reference)		1.00 (reference)		1.00 (reference)		1.00 (reference)	
Diabetes	1.21 (1.08, 1.34)	<0.001	0.98 (0.88, 1.09)	0.680	1.34 (1.25, 1.44)	<0.001	1.09 (1.02, 1.17)	0.012	1.31 (1.23, 1.40)	<0.001	1.08 (1.01, 1.15)	0.033
**History of Coronary Heart Disease (CHD) (as of 1/1/1999)**												
No	1.00 (reference)		1.00 (reference)		1.00 (reference)		1.00 (reference)		1.00 (reference)		1.00 (reference)	
Yes	0.92 (0.80, 1.06)	0.266	0.94 (0.81, 1.08)	0.370	0.95 (0.86, 1.04)	0.263	0.96 (0.87, 1.06)	0.420	0.94 (0.86, 1.03)	0.206	0.96 (0.87, 1.05)	0.330
**Atherosclerotic Cardiovascular Disease (ASCVD) at trial baseline**												
No	1.00 (reference)		1.00 (reference)		1.00 (reference)		1.00 (reference)		1.00 (reference)		1.00 (reference)	
Yes	1.18 (1.00, 1.38)	0.046	1.21 (1.03, 1.42)	0.018	1.14 (1.03, 1.26)	0.015	1.16 (1.05, 1.29)	0.005	1.16 (1.04, 1.28)	0.005	1.18 (1.07, 1.31)	0.001
**History of myocardial infarction (MI) or stroke (as of 1/1/1999)**												
No	1.00 (reference)		1.00 (reference)		1.00 (reference)		1.00 (reference)		1.00 (reference)		1.00 (reference)	
Yes	1.07 (0.93, 1.24)	0.344	0.89 (0.77, 1.02)	0.096	1.22 (1.11, 1.34)	<0.001	1.01 (0.92, 1.11)	0.830	1.21 (1.11, 1.33)	<0.001	1.01 (0.92, 1.11)	0.800
**History of coronary artery bypass graft (CABG) (as of 1/1/1999)**												
No	1.00 (reference)		1.00 (reference)		1.00 (reference)		1.00 (reference)		1.00 (reference)		1.00 (reference)	
Yes	0.95 (0.80, 1.13)	0.573	0.87 (0.73, 1.03)	0.120	1.02 (0.92, 1.14)	0.716	0.94 (0.84, 1.04)	0.240	1.00 (0.90, 1.11)	0.946	0.93 (0.83, 1.03)	0.160
**Other ASCVD at trial baseline**												
No	1.00 (reference)		1.00 (reference)		1.00 (reference)		1.00 (reference)		1.00 (reference)		1.00 (reference)	
Yes	0.96 (0.83, 1.11)	0.615	0.88 (0.77, 1.02)	0.099	1.04 (0.94, 1.14)	0.442	0.95 (0.86, 1.04)	0.250	1.02 (0.93, 1.11)	0.715	0.93 (0.85, 1.02)	0.120
**Major ST segment depression (as of 1/1/1999)**												
No	1.00 (reference)		1.00 (reference)		1.00 (reference)		1.00 (reference)		1.00 (reference)		1.00 (reference)	
Yes	0.94 (0.77, 1.15)	0.542	0.83 (0.68, 1.01)	0.057	0.96 (0.84, 1.09)	0.529	0.84 (0.73, 0.95)	0.008	0.95 (0.83, 1.08)	0.422	0.83 (0.73, 0.95)	0.005
**Left ventricular hypertrophy (LVH) by Minnesota code (as of 1/1/1999)**												
No/Soft LVH	1.00 (reference)		1.00 (reference)		1.00 (reference)		1.00 (reference)		1.00 (reference)		1.00 (reference)	
Hard LVH	1.07 (0.82, 1.38)	0.624	0.88 (0.68, 1.14)	0.330	1.11 (0.94, 1.32)	0.220	0.93 (0.78, 1.11)	0.420	1.13 (0.96, 1.33)	0.127	0.95 (0.80, 1.13)	0.580
**Lipid Lowering Trial (LLT) Participant**												
No	1.00 (reference)		1.00 (reference)		1.00 (reference)		1.00 (reference)		1.00 (reference)		1.00 (reference)	
Yes	0.94 (0.84, 1.05)	0.266	0.97 (0.87, 1.09)	0.630	0.95 (0.88, 1.02)	0.188	0.98 (0.91, 1.06)	0.640	0.94 (0.88, 1.01)	0.107	0.97 (0.90, 1.05)	0.460
**Obesity (BMI ≥ 30 kg/m²) at trial baseline**												
No	1.00 (reference)		1.00 (reference)		1.00 (reference)		1.00 (reference)		1.00 (reference)		1.00 (reference)	
Yes	0.84 (0.76, 0.94)	0.001	0.84 (0.76, 0.93)	<0.001	1.02 (0.95, 1.09)	0.589	1.01 (0.95, 1.08)	0.730	0.98 (0.92, 1.05)	0.569	0.97 (0.91, 1.04)	0.420
**Blood pressure change (latest BP reading prior to 1/1/1999 minus BP at trial baseline), per 10 mmHg**												
Systolic BP	0.99 (0.96, 1.03)	0.655	0.99 (0.96, 1.02)	0.590	1.00 (0.98, 1.03)	0.680	1.00 (0.98, 1.03)	0.680	1.00 (0.98, 1.02)	0.816	1.00 (0.98, 1.02)	0.830
Diastolic BP	1.00 (0.95, 1.06)	0.919	1.01 (0.95, 1.07)	0.830	1.03 (0.99, 1.07)	0.161	1.03 (0.99, 1.07)	0.130	1.04 (1.00, 1.07)	0.057	1.04 (1.00, 1.08)	0.046

ADRD= Alzheimer's Disease and Related Dementias; HR= Hazard Ratio; CI= Confidence Interval**;** CRR= Competing Risks Regression

* Adjusted for each covariate shown, in addition to all others presented in the table.

‡Contrast estimates were garnered from the same model, using a different reference group for randomized group (Chlorthalidone or Amlodipine).

†Estrogen use was evaluated in women only, which prevented simultaneous inclusion of sex and estrogen as covariates

Table S4: Hazard ratio (HR) of Primary Diagnosis of ADRD (with ≥2 occurrences with 30-day period of separation) by Randomized Group (3 Study Drugs) and Other Factors (1999 to 2017), Calculated by Standard Cox Proportional Hazards Model or by Competing Risks Regression (CRR) Model

Demographic	Alzheimer's Disease (AD)				Other non-AD Dementias				All ADRD combined			
	Adjusted Standard HR (95% CI)	P value	Adjusted CRR HR (95% CI)	P value	Adjusted Standard HR (95% CI)	P value	Adjusted CRR HR (95% CI)	P value	Adjusted Standard HR (95% CI)	P value	Adjusted CRR HR (95% CI)	P value
**Randomized group**												
Chlorthalidone vs Amlodipine	1.11 (0.94, 1.32)	0.207	1.09 (0.92, 1.29)	0.310	0.95 (0.85, 1.05)	0.321	0.93 (0.84, 1.03)	0.170	0.96 (0.87, 1.06)	0.420	0.94 (0.85, 1.04)	0.230
Lisinopril vs Amlodipine	1.04 (0.86, 1.26)	0.667	1.03 (0.85, 1.25)	0.780	0.99 (0.88, 1.11)	0.814	0.98 (0.87, 1.10)	0.680	1.00 (0.89, 1.11)	0.956	0.98 (0.88, 1.10)	0.760
Lisinopril vs Chlorthalidone‡	0.94 (0.79, 1.11)	0.441	0.94 (0.80, 1.12)	0.490	1.04 (0.93, 1.16)	0.473	1.05 (0.94, 1.17)	0.380	1.04 (0.94, 1.14)	0.462	1.04 (0.95, 1.15)	0.400
**Age group (as of 1/1/1999)**												
Age <70	1.00 (reference)		1.00 (reference)		1.00 (reference)		1.00 (reference)		1.00 (reference)		1.00 (reference)	
Age 70-79	2.10 (1.77, 2.49)	<0.001	1.71 (1.44, 2.02)	<0.001	1.72 (1.55, 1.91)	<0.001	1.39 (1.26, 1.54)	<0.001	1.77 (1.61, 1.95)	<0.001	1.45 (1.32, 1.59)	<0.001
Age 80+	3.26 (2.61, 4.08)	<0.001	1.62 (1.30, 2.02)	<0.001	3.23 (2.82, 3.70)	<0.001	1.62 (1.42, 1.86)	<0.001	3.30 (2.91, 3.74)	<0.001	1.72 (1.52, 1.94)	<0.001
**Gender**												
Male	1.00 (reference)		1.00 (reference)		1.00 (reference)		1.00 (reference)		1.00 (reference)		1.00 (reference)	
Female	1.24 (1.05, 1.46)	0.012	1.36 (1.16, 1.60)	<0.001	1.10 (0.99, 1.22)	0.063	1.20 (1.09, 1.33)	<0.001	1.17 (1.06, 1.28)	0.001	1.27 (1.16, 1.40)	<0.001
**Race/Ethnicity**												
Non-Black	1.00 (reference)		1.00 (reference)		1.00 (reference)		1.00 (reference)		1.00 (reference)		1.00 (reference)	
Black	1.09 (0.93, 1.28)	0.268	1.11 (0.95, 1.30)	0.190	1.06 (0.96, 1.18)	0.228	1.08 (0.98, 1.19)	0.130	1.07 (0.97, 1.17)	0.173	1.08 (0.99, 1.19)	0.097
**Hispanic/Latino Ethnicity**												
Non-Hispanic	1.00 (reference)		1.00 (reference)		1.00 (reference)		1.00 (reference)		1.00 (reference)		1.00 (reference)	
Hispanic	0.85 (0.70, 1.02)	0.087	0.93 (0.77, 1.12)	0.450	0.43 (0.37, 0.50)	<0.001	0.47 (0.41, 0.55)	<0.001	0.58 (0.52, 0.66)	<0.001	0.64 (0.57, 0.73)	<0.001
**Education level**												
>High school	1.00 (reference)		1.00 (reference)		1.00 (reference)		1.00 (reference)		1.00 (reference)		1.00 (reference)	
≤High school	1.12 (0.95, 1.32)	0.182	1.02 (0.87, 1.21)	0.800	1.04 (0.94, 1.15)	0.504	0.94 (0.85, 1.05)	0.270	1.03 (0.94, 1.14)	0.500	0.94 (0.86, 1.04)	0.230
**Treatment with antihypertensive drugs prior to trial baseline**												
Untreated	1.00 (reference)		1.00 (reference)		1.00 (reference)		1.00 (reference)		1.00 (reference)		1.00 (reference)	
Treated	0.82 (0.66, 1.03)	0.091	0.80 (0.64, 1.00)	0.053	0.92 (0.79, 1.06)	0.249	0.89 (0.76, 1.03)	0.110	0.92 (0.80, 1.05)	0.213	0.89 (0.77, 1.02)	0.088
**Aspirin use (as of 1/1/1999)**												
No	1.00 (reference)		1.00 (reference)		1.00 (reference)		1.00 (reference)		1.00 (reference)		1.00 (reference)	
Yes	0.94 (0.81, 1.10)	0.465	0.96 (0.82, 1.12)	0.630	0.94 (0.85, 1.03)	0.200	0.96 (0.87, 1.06)	0.380	0.92 (0.84, 1.01)	0.066	0.93 (0.85, 1.02)	0.140
**HDL cholesterol <35 mg/dl (as of 1/1/1999)**												
No	1.00 (reference)		1.00 (reference)		1.00 (reference)		1.00 (reference)		1.00 (reference)		1.00 (reference)	
Yes	0.83 (0.66, 1.04)	0.106	0.79 (0.63, 0.99)	0.038	0.92 (0.80, 1.05)	0.193	0.87 (0.77, 1.00)	0.046	0.92 (0.82, 1.05)	0.211	0.88 (0.78, 1.00)	0.044
**Cigarette smoking at trial baseline**												
Never smoker	1.00 (reference)		1.00 (reference)		1.00 (reference)		1.00 (reference)		1.00 (reference)		1.00 (reference)	
Current smoker	0.76 (0.59, 0.97)	0.029	0.56 (0.44, 0.71)	<0.001	1.02 (0.89, 1.18)	0.757	0.75 (0.65, 0.87)	<0.001	1.00 (0.88, 1.15)	0.953	0.74 (0.65, 0.85)	<0.001
Former smoker	0.93 (0.79, 1.09)	0.344	0.85 (0.73, 1.00)	0.052	0.94 (0.85, 1.04)	0.202	0.87 (0.78, 0.96)	0.006	0.94 (0.86, 1.03)	0.181	0.87 (0.79, 0.96)	0.004
**Diabetes classification (as of 1/1/1999)**												

Non-diabetes	1.00 (reference)		1.00 (reference)		1.00 (reference)		1.00 (reference)		1.00 (reference)		1.00 (reference)	
Diabetes	1.14 (0.98, 1.33)	0.091	0.93 (0.80, 1.08)	0.340	1.24 (1.13, 1.36)	<0.001	1.01 (0.92, 1.11)	0.810	1.25 (1.14, 1.37)	<0.001	1.03 (0.94, 1.12)	0.590
**History of Coronary Heart Disease (CHD) (as of 1/1/1999)**												
No	1.00 (reference)		1.00 (reference)		1.00 (reference)		1.00 (reference)		1.00 (reference)		1.00 (reference)	
Yes	1.05 (0.86, 1.29)	0.603	1.07 (0.88, 1.30)	0.510	1.02 (0.90, 1.17)	0.714	1.04 (0.92, 1.19)	0.510	1.04 (0.92, 1.17)	0.555	1.05 (0.94, 1.19)	0.390
**Atherosclerotic Cardiovascular Disease (ASCVD) at trial baseline**												
No	1.00 (reference)		1.00 (reference)		1.00 (reference)		1.00 (reference)		1.00 (reference)		1.00 (reference)	
Yes	1.32 (1.05, 1.66)	0.016	1.35 (1.09, 1.68)	0.007	0.97 (0.84, 1.12)	0.646	0.99 (0.86, 1.14)	0.910	1.04 (0.91, 1.19)	0.564	1.07 (0.93, 1.22)	0.340
**History of myocardial infarction (MI) or stroke (as of 1/1/1999)**												
No	1.00 (reference)		1.00 (reference)		1.00 (reference)		1.00 (reference)		1.00 (reference)		1.00 (reference)	
Yes	0.96 (0.78, 1.17)	0.659	0.80 (0.66, 0.97)	0.023	1.21 (1.06, 1.37)	0.004	1.00 (0.88, 1.13)	0.980	1.15 (1.03, 1.30)	0.016	0.96 (0.85, 1.08)	0.470
**History of coronary artery bypass graft (CABG) (as of 1/1/1999)**												
No	1.00 (reference)		1.00 (reference)		1.00 (reference)		1.00 (reference)		1.00 (reference)		1.00 (reference)	
Yes	0.94 (0.74, 1.19)	0.591	0.86 (0.68, 1.08)	0.190	1.03 (0.89, 1.19)	0.724	0.95 (0.82, 1.10)	0.470	1.01 (0.88, 1.16)	0.862	0.94 (0.82, 1.07)	0.350
**Other ASCVD at trial baseline**												
No	1.00 (reference)		1.00 (reference)		1.00 (reference)		1.00 (reference)		1.00 (reference)		1.00 (reference)	
Yes	0.84 (0.68, 1.03)	0.089	0.77 (0.63, 0.95)	0.013	1.08 (0.95, 1.22)	0.271	0.99 (0.87, 1.12)	0.850	1.04 (0.92, 1.17)	0.569	0.95 (0.85, 1.07)	0.420
**Major ST segment depression (as of 1/1/1999)**												
No	1.00 (reference)		1.00 (reference)		1.00 (reference)		1.00 (reference)		1.00 (reference)		1.00 (reference)	
Yes	0.87 (0.65, 1.17)	0.352	0.78 (0.59, 1.03)	0.076	1.01 (0.84, 1.21)	0.898	0.89 (0.74, 1.06)	0.200	0.97 (0.82, 1.14)	0.705	0.86 (0.73, 1.01)	0.064
**Left ventricular hypertrophy (LVH) by Minnesota code (as of 1/1/1999)**												
No/Soft LVH	1.00 (reference)		1.00 (reference)		1.00 (reference)		1.00 (reference)		1.00 (reference)		1.00 (reference)	
Hard LVH	0.75 (0.49, 1.16)	0.198	0.62 (0.41, 0.95)	0.028	1.07 (0.85, 1.35)	0.562	0.90 (0.71, 1.15)	0.400	1.05 (0.85, 1.31)	0.629	0.88 (0.71, 1.10)	0.270
**Lipid Lowering Trial (LLT) Participant**												
No	1.00 (reference)		1.00 (reference)		1.00 (reference)		1.00 (reference)		1.00 (reference)		1.00 (reference)	
Yes	0.97 (0.83, 1.14)	0.752	1.01 (0.86, 1.19)	0.900	0.97 (0.87, 1.07)	0.510	1.00 (0.91, 1.11)	0.960	0.94 (0.85, 1.03)	0.171	0.97 (0.89, 1.07)	0.600
**Obesity (BMI ≥ 30 kg/m²) at trial baseline**												
No	1.00 (reference)		1.00 (reference)		1.00 (reference)		1.00 (reference)		1.00 (reference)		1.00 (reference)	
Yes	0.75 (0.65, 0.88)	<0.001	0.75 (0.65, 0.87)	<0.001	1.00 (0.91, 1.10)	0.980	1.00 (0.91, 1.10)	0.970	0.92 (0.85, 1.01)	0.068	0.92 (0.84, 1.00)	0.058
**Blood pressure change (latest BP reading prior to 1/1/1999 minus baseline), per 10 mmHg**												
Systolic BP	1.01 (0.96, 1.05)	0.815	1.00 (0.96, 1.05)	0.880	1.00 (0.97, 1.03)	0.966	1.00 (0.97, 1.03)	0.880	1.00 (0.97, 1.02)	0.804	1.00 (0.97, 1.02)	0.720
Diastolic BP	0.96 (0.89, 1.05)	0.375	0.97 (0.89, 1.06)	0.490	1.00 (0.95, 1.05)	0.981	1.00 (0.95, 1.06)	0.860	1.01 (0.96, 1.06)	0.687	1.01 (0.96, 1.06)	0.590

ADRD= Alzheimer's Disease and Alzheimer's Disease Related Dementias; HR= Hazard Ratio; CI= Confidence Interval; CRR= Competing Risks Regression

* Adjusted for each covariate shown, in addition to all others presented in the table.

‡Contrast estimates were garnered from the same model, using a different reference group for randomized group (Chlorthalidone or Amlodipine).

†Estrogen use was evaluated in women only, which prevented simultaneous inclusion of sex and estrogen as covariates

Methods for identifying the posttrial antihypertensive medications from Medicare Part-D Data

These post-trial antihypertensive drugs for those subjects enrolled in Medicare Part-D comprehensive drug program in 2007-2017 were identified from Medicare Part-D data through brand or generic drug names or the National Drug Codes of antihypertensive drugs, which were then classified into 16 major categories (see Supplement Table S1 and the following published study[1] for details).

Reference: 1. Du XL, Simpson LM, Tandy BC, Bettencourt JL, Davis BR. Effects of Posttrial Antihypertensive Drugs on Morbidity and Mortality: Findings from 15-Year Passive Follow-Up after ALLHAT Ended. *International Journal of Hypertension*. 2021 Dec 9; ID 2261144;15 pages. https://doi.org/10.1155/2021/2261144.

Table S5. Adjusted* Hazard Ratios for Cumulative Incidence of ADRD by Categories of Post-trial Antihypertensive Drug Uses from Medicare Part-D Drug Data in 2007-2017, Stratified by the 3 Study Drugs (Total Participants: N=6086)

**Diuretic= Thiazide/thiazide-type** [Randomized to Chlorthalidone in trial: 2766 (45.4)]	**Participants who were randomized to a diuretic and continued to receive a diuretic post-trial (N=1323)**				**Participants who were randomized to a diuretic and did not receive a diuretic post-trial (N=1443)**				
	1) any diuretic (1 drug) (N=67)	2) any diuretic plus 1 drug (2 drugs) (N=309)	3) any diuretic plus 2 drugs (3 drugs) (N=435)	4) any diuretic plus ≥3 drugs (4+ drugs) (N=512)	1) no diuretic (on 1 drug) (N=286)	2) no diuretic (on 2 drugs) (N=406)	3) no diuretic (on 3 drugs) (N=327)	4) no diuretic (on 4+ drugs) (N=277)	5) No Drugs (N=147)
Alzheimer's Disease	1.00 (ref)	0.89 (0.39,2.05)	0.69 (0.30,1.59)	0.85 (0.37,1.91)	1.38 (0.61,3.15)	1.16 (0.51,2.61)	1.12 (0.48,2.60)	1.40 (0.61,3.24)	1.71 (0.71,4.11)
Any ADRD	1.00 (ref)	0.83 (0.49,1.41)	0.86 (0.51,1.45)	0.99 (0.59,1.65)	1.06 (0.62,1.81)	1.04 (0.62,1.76)	1.14 (0.67,1.93)	1.21 (0.71,2.08)	1.20 (0.67,2.14)
Non-Alzheimer's Dementia	1.00 (ref)	0.91 (0.52,1.60)	0.97 (0.56,1.68)	1.11 (0.65,1.91)	1.17 (0.67,2.06)	1.16 (0.67,2.01)	1.28 (0.73,2.24)	1.31 (0.74,2.31)	1.23 (0.67,2.26)
**Calcium channel blocker (CCB)** [Randomized to Amlodipine in trial: 1650 (27.1)]	**Participants who were randomized to a CCB and continued to receive a CCB post-trial (N=871)**				**Participants who were randomized to a CCB and did not receive a CCB post-trial (N=779)**				
	1) any CCB (1 drug) (N=75)	2) any CCB plus 1 drug (2 drugs) (N=175)	3) any CCB plus 2 drugs (3 drugs) (N=250)	4) any CCB plus ≥3 drugs (4+ drugs) (N=371)	1) no CCB (on 1 drug) (N=164)	2) no CCB (on 2 drugs) (N=253)	3) no CCB (on 3 drugs) (N=188)	4) no CCB (on 4+ drugs) (N=82)	5) No Drugs (N=92)
Alzheimer's Disease	1.00 (ref)	0.57 (0.25,1.29)	0.65 (0.30,1.38)	0.68 (0.33,1.41)	1.07 (0.50,2.32)	0.76 (0.36,1.60)	0.53 (0.23,1.21)	0.82 (0.31,2.19)	0.63 (0.24,1.63)
Any ADRD	1.00 (ref)	1.21 (0.71,2.05)	1.27 (0.76,2.12)	1.25 (0.76,2.05)	1.29 (0.75,2.23)	1.29 (0.78,2.16)	1.15 (0.67,1.97)	1.33 (0.70,2.53)	1.20 (0.65,2.22)
Non-Alzheimer's Dementia	1.00 (ref)	1.19 (0.69,2.05)	1.29 (0.77,2.18)	1.31 (0.79,2.18)	1.32 (0.75,2.31)	1.33 (0.79,2.25)	1.14 (0.65,1.97)	1.43 (0.75,2.75)	1.15 (0.61,2.17)
**Angiotensin-converting enzyme (ACE) inhibitor / Angiotensin II receptor blockers (ARB)** [Randomized to Lisinopril in trial: 1670 (27.4)]	**Participants who were randomized to an ACE/ARB and continued to receive an ACE/ARB post-trial (N=1216)**				**Participants who were randomized to an ACE/ARB and did not receive an ACE/ARB post-trial (N=454)**				
	1) any ACE/ARB (1 drug) (N=94)	2) any ACE/ARB plus 1 drug (2 drugs) (N=290)	3) any ACE/ARB plus 2 drugs (3 drugs) (N=412)	4) any ACE/ARB plus ≥3 drugs (4+ drugs) (N=420)	1) no ACE/ARB (on 1 drug) (N=135)	2) no ACE/ARB (on 2 drugs) (N=115)	3) no ACE/ARB (on 3 drugs) (N=86)	4) no ACE/ARB (on 4+ drugs) (N=35)	5) No Drugs (N=83)
Alzheimer's Disease	1.00 (ref)	0.83 (0.39,1.78)	0.91 (0.44,1.91)	1.01 (0.48,2.10)	0.83 (0.33,2.05)	0.98 (0.39,2.43)	1.49 (0.62,3.58)	0.94 (0.25,3.54)	1.10 (0.39,3.13)
Any ADRD	1.00 (ref)	0.80 (0.48,1.34)	1.18 (0.73,1.90)	1.31 (0.81,2.13)	0.97 (0.53,1.77)	1.32 (0.75,2.35)	1.13 (0.61,2.08)	1.70 (0.82,3.53)	0.87 (0.42,1.83)
Non-Alzheimer's Dementia	1.00 (ref)	0.84 (0.50,1.41)	1.19 (0.72,1.94)	1.37 (0.84,2.25)	0.88 (0.47,1.65)	1.40 (0.78,2.50)	1.05 (0.55,1.98)	1.83 (0.87,3.83)	0.74 (0.33,1.65)

*Baseline covariates in the adjusted hazard ratio models include: Age as of 1/1/2007 (years), race (Black/Non-Black), gender, education (High School or less/More than High School), smoking status at trial baseline, BMI at trial baseline, HDL<35 as of 1/1/2007, LDL as of 1/1/2007, history of diabetes as of 1/1/2007, antihypertensive treatment at trial entry, mean systolic blood pressure (SBP) as of 1/1/2007.
